# The impact of immunotherapies on COVID-19 case fatality rates during the US vaccination campaign: a multidisciplinary open data analysis using FDA Adverse Event Reporting System and Our World in Data

**DOI:** 10.3389/fphar.2023.1186404

**Published:** 2023-06-15

**Authors:** Anke Salmen, Stefanie Marti, Andreas G. F. Hoepner, Andrew Chan, Robert Hoepner

**Affiliations:** ^1^ Department of Neurology, Inselspital, Bern University Hospital and University of Bern, Bern, Switzerland; ^2^ Department of Banking and Finance, Michael Smurfit Graduate Business School, University College Dublin, Dublin, Ireland; ^3^ Department for Financial Stability and Capital Markets (DG FISMA), Platform for Sustainable Finance, European Commission, Brussels, Belgium

**Keywords:** COVID-19, SARS-CoV-2, mortality, immunotherapy, FAERS, our world in data, vaccination SARS-CoV-2

## Abstract

**Introduction:** Patients under immunotherapies were excluded from the pivotal trials of vaccinations against the severe acute respiratory syndrome coronavirus type 2 (SARS-CoV-2), and no population-level data on disease outcomes such as case fatality rates in relation to vaccination coverage exist. Our study aims to fill this gap by investigating whether CFRs in patients with immunotherapies decrease with increasing vaccination coverage in the total population.

**Methods:** We combined aggregated open source data on COVID-19 vaccination coverage from “Our World in Data” with publicly available anonymized COVID-19 case reports from the FDA Adverse Event Reporting System to compute COVID-19 CFRs for patients under immunotherapy at different vaccination coverage levels in the total population. CFRs at different vaccination coverage levels were then compared to CFRs before vaccination campaign start.

**Results:** While we found an overall decrease in CFRs on population level with increasing vaccination coverage, we found no decrease in people using anti-CD20 or glucocorticoids.

**Discussion:** Risk-mitigation strategies on an individual- and population-level are thus still needed to lower the probability of fatal SARS-CoV2 infection for these vulnerable populations.

## 1 Introduction

End of 2019, a pandemic with the severe acute respiratory syndrome coronavirus type 2 (SARS-CoV-2) emerged, which, as of 9 October 2022, has led to more than 626 million cases and 6 million deaths worldwide, corresponding to a case fatality rate (CFR) of 1.047% (Worldometers.info, 2022).

During the first year of the pandemic, non-pharmaceutical interventions such as restrictions in gatherings or the mandatory use of face masks were the main instruments to control the pandemic spread and to reduce death rates (Sonabend et al., 2021; Yuan and Blakemore, 2022). However, since 8 December 2020, the date of the first use of an anti-SARS-CoV2 mRNA vaccination worldwide (NHS England, 2020), vaccination strategies have gained importance. Initially, vaccinations were mainly available for population groups at higher risk of severe Corona Virus Disease-19 (COVID-19). Today, vaccination is recommended by the US Food and Drug Administration (FDA) for the general population older than 6 months of age (U.S. Food & Drug Administration FDA, 2022). Despite those initial limitations in vaccination availability, already in the first year after market launch SARS-CoV-2 vaccinations have saved an estimated number of 14.4 million lives worldwide (Watson et al., 2022).

Medical research has focused on identifying predisposing factors associated with a decreased response to vaccination. In addition to person-sided factors such as age, sex, and obesity (Falahi and Kenarkoohi, 2022), the impact of immunotherapies has been studied. While some studies found no significant impact of immunotherapies on vaccination efficacy as measured by humoral immune and T cell response (Dimopoulou et al., 2022; Venerito et al., 2022), others found a considerable reduction (Moor et al., 2021; Wu et al., 2022).

This population group of pharmacologically immunosuppressed persons—though representing approximately 9 million people in the United States (Harpaz et al., 2013; United States Census Bureau, 2020),—was excluded from the pivotal phase 3 trials of the two mRNA vaccines (Polack et al., 2020; Baden et al., 2021). Further, studies in people under certain immunotherapies investigated vaccination efficacy on an individual case-level using humoral immune response and T cell response as proxies of efficacy for an expected poorer outcome of a SARS-CoV-2 infection.

Thus, the knowledge gap of the burden of immunotherapies on CFRs after introduction of vaccination strategies remains open. This study aims to answer this question on a population-level using the publicly available data sources FDA Adverse Event Reporting System (FAERS) (U.S. Food & Drug Administration FDA, 2012) and Our World in Data (OWID) (Mathieu et al., 2020) by linking immunotherapies to CFRs as a function of vaccination rates.

## 2 Methods

### 2.1 Study design

We combined population-level data on COVID-19 vaccination rates from OWID (Mathieu et al., 2020) with data from individual COVID-19 case reports submitted to FAERS (U.S. Food & Drug Administration FDA, 2012), a post-marketing, self-reporting open-access pharmacovigilance platform, to investigate whether an increase of the vaccination coverage in the total population is associated with a decrease in COVID-19 CFRs in patients under different immunotherapies irrespective of their individual vaccination status.

We compared the rate of deaths in COVID-19 case reports from FAERS before the start of the vaccination campaign to the corresponding rate after a certain proportion of the total population had received at least one vaccination dose. The analysis was performed on country-level to avoid confounding due to different courses of the pandemic in different countries, e.g., due to differences in the onset of variant waves relative to the vaccination rate, differences in mitigation strategies and political decisions on non-pharmaceutical interventions, or healthcare accessibility.

### 2.2 Data

We downloaded all reports with a COVID-19 term in *Reactions* from FAERS (U.S. Food & Drug Administration FDA, 2012) for individual COVID-19 case reports and applied a stepwise filtering procedure to build datasets with enough reports for a robust statistical analysis (see *Data selection* in Supplementary methods for details on data selection).

Only data from the United States (49,742 case reports mentioning COVID-19 as a reaction, of which 3,756 cases with death as reported outcome), and only the treatment groups anti-Cluster of Differentiation (CD) 20 (1,907 cases, 202 deaths), anti-Tumor Necrosis Factor (TNF) α (3,189 cases, 98 deaths), glucocorticoids (1,176 cases, 208 deaths), Janus Kinase (JAK) inhibitors (3,572 cases, 183 deaths), and thalidomide analogs (4,307 cases, 424 deaths) fulfilled our filter criteria (see Table 1; Supplementary Table S1 for cohort characteristics).

**TABLE 1:** COVID-19 cases from FAERS reports, US only, overall and by treatment group. COVID-19 cases are all cases reported to FAERS with a COVID-19 related term in *Reactions*. Treatment groups are monotherapy only except for glucocorticoids, but within-group combinations (e.g., Ocrelizumab + Rituximab) are included. Cases with COVID-19 as only indication are excluded from the treatment group data sets. Data from 2020–01–22 to 2022–06–30, US. Cases are counted as *died* if death was reported in the outcomes list, and as *survived* otherwise. The lines printed in bold are group totals/group headers. Note that since within-group combinations are allowed, the sum over all cases for all individual treatments within a treatment group may be greater than the total number of cases for the entire treatment group. ***Abbreviations:*** IQR: Interquartile range, the 25^th^ and 75^th^ percentile; COVID-19: Corona Virus Disease-2019; FAERS: FDA Adverse Event Reporting System.

	Overall	Died	Survived
**Whole dataset**: All COVID-19 cases in FAERS, US	**49,742**	**3756**	**45,986**
**Sex (n, %)**			
Female	28,587 (57.47)	1434 (38.18)	27,153 (59.05)
Male	17,421 (35.02)	1897 (50.51)	15,524 (33.76)
Not specified	3734 (7.51)	425 (11.32)	3309 (7.20)
**Age (Median, IQR)**			
**Overall**	**59 (47–69)**	**70 (60–78)**	**58 (46–67)**
Female	58 (46–67)	68 (60–78)	57 (46–67)
Male	61 (48–70)	70 (62–78)	59 (46–69)
Not specified	62 (45–70)	65 (54–71)	60 (44–69)
**Treatment subgroups**: COVID-19 cases in FAERS, US, by treatment			
**Anti-CD20, monotherapy**	**1907**	**202**	**1705**
Ocrelizumab	1142	91	1051
Ofatumumab	459	8	451
Rituximab	320	104	216
**Anti-TNFα, monotherapy**	**3189**	**98**	**3091**
Adalimumab	1807	65	1742
Certolizumab Pegol	625	12	613
Etanercept	597	11	586
Golimumab	78	1	77
Infliximab	96	9	87
**Glucocorticoids**	**1176**	**208**	**968**
Betamethasone	8	0	8
Cortisone	1	0	1
Dexamethasone	186	57	129
Dexamethasone Sodium Phosphate	7	5	2
Methylprednisolone	76	12	64
Methylprednisolone Sodium Succinate	15	9	6
Prednisolone	158	25	133
Prednisone	811	131	680
**JAK inhibitors, monotherapy**	**3572**	**183**	**3389**
Baricitinib	53	16	37
Ruxolitinib	396	64	332
Tofacitinib Citrate	2439	56	2383
Upadacitinib	686	47	639
**Thalidomide analogs, monotherapy**	**4307**	**424**	**1835**
Lenalidomide	3350	337	3013
Pomalidomide	934	80	854
Thalidomide	54	13	41

Note that cases where the indication *Reason for Use* only contains COVID-19 terms were excluded from the treatment groups to mitigate the potential bias from confounding by indication, and except for the glucocorticoid group, where all cases were included irrespective of concomitant treatments, only monotherapy cases, i.e., cases where only treatments for a given group were listed under *Suspect Product Active Ingredient*, were included (see Data selection in Supplementary methods for details on data selection, and Table 1; Supplementary Table S1 for cohort characteristics).

### 2.3 Statistical analysis

COVID-19 case reports from FAERS were combined into bins based on the percentage of the total population who had received at least one vaccination dose at the time of reporting (see Supplementary Table S2 for details on how the bins were defined). For each bin and the baseline period, defined as the period before the first vaccination dose was administered, we computed CFRs defined as the number of COVID-19 case reports with death as reported outcome divided by the total number of COVID-19 case reports submitted within the respective period. We estimated 95% confidence intervals (95%CI) for the CFRs using bootstrap resampling (9,999 resamples, bias corrected and accelerated method). For the OWID dataset, we used Wilson’s score interval since bootstrapping was not feasible due to large case numbers.

For each bin, we compared the CFR for this period to the CFR of the baseline period of the corresponding dataset, and tested the observed difference against the null hypothesis that there is no difference in COVID-19 CFRs between the bin’s period and the baseline period using a permutation test (9,999 random resamples, two-sided test). For OWID data, where sample sizes are too large for a resampling test, we used the G-test instead. We did not compare different treatment groups among each other since we cannot infer any absolute estimates of mortality from FAERS reports (see discussion section).

To account for multiple comparisons in significance testing, we employed the Benjamini-Hochberg procedure with an accepted false discovery rate (FDR) of 5% over all bins and treatment groups.

#### 2.3.1 Assessment of FAERS data quality and analysis of potential biases

To assess whether FAERS data can serve as a reasonable proxy for the total population, and to assess potential confounders, we also analyzed whether the CFR derived from all FAERS COVID-19 case reports follows the CFR from OWID, whether the known risk factors age and male sex (Falahi and Kenarkoohi, 2022) are reflected in FAERS data, whether the sex and age distributions pre- and post-campaign start are comparable, and whether the prioritization of people over 65 years for vaccination is reflected in FAERS data from the United States.

#### 2.3.2 Software

We did all data preprocessing and analyses in Python 3.9.7 using scipy. stats for bootstrapping, permutation tests, and the G-test, and the matplotlib and seaborn packages for visualization.

## 3 Results

### 3.1 FAERS COVID-19 data quality assessments

#### 3.1.1 FAERS-derived CFRs compared to OWID-derived CFRs

**FIGURE 1:**
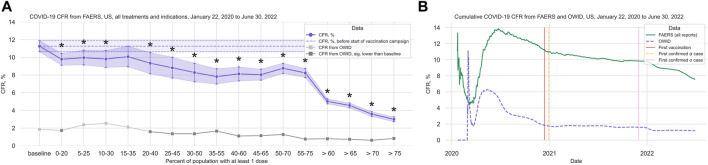
COVID-19 CFR from FAERS and from OWID, US. **(A)** CFR in percent computed for the complete FAERS COVID-19 dataset for the US (all patients with COVID-19, irrespective of treatment or indication) during the baseline period (first bin, and dashed line with 95% confidence interval drawn over the entire plot range for reference) and for 20% bins in vaccination coverage (at least one dose). Bins are indicated as (lower bound, upper bound], and the baseline period is defined as the period before the first vaccination was administered. Since a coverage of 80% had not yet been achieved at the time of data collection, we denote bins starting from 60% or higher coverage as “> *x*,” indicating that these bins include all data from coverage levels higher than x. The 95% confidence intervals were estimated using bootstrap resampling, and asterisks mark data points where the CFR is significantly different from the CFR during the baseline period (*p*-value from resampling, Benjamini-Hochberg with an accepted FDR of 5% over all bins and treatment groups to adjust for multiple testing). The grey line shows the CFR for OWID data for reference, with data points where the CFR is significantly lower than during the baseline period shown in a darker grey. **(B)** Cumulative CFR from OWID data (blue dashed line) and the entire FAERS COVID-19 dataset (all patients with COVID-19, irrespective of treatments or indications, green solid line), for the US. The first vaccination was administered on 13 December 2020 (vertical solid red line), the first Alpha variant case was confirmed on 29 December 2020 (vertical dot-dashed orange line), and the first Omicron variant case was confirmed on 1 December 2021 (vertical dotted pink line). Abbreviations: CFR: Case Fatality Rate; COVID-19: Corona Virus Disease-2019; FAERS: FDA Adverse Event Reporting System; OWID: Our World In Data.

Both the CFR computed from OWID data (total 87,675,711 cases, 1,018,576 deaths; Supplementary Figure S1; Supplementary Table S3) and the CFR computed from all FAERS COVID-19 cases (total 49,742 cases, 3,756 deaths) decrease with increasing vaccination coverage. We observed a strong decrease in the rate of reported deaths in FAERS after 60% of the population had received at least one vaccination dose (Figure 1A; Supplementary Table S4).

We observed that the cumulative CFR computed from all COVID-19-related reports in FAERS follows the CFR computed from OWID data qualitatively with a slight delay (Figure 1B). This observed delay is due to different date sources in OWID and FAERS data: While for OWID data the actual event date is available, we have to use the report date for FAERS data since the actual event date is only specified in 17,004 (33.30%) of all 51,070 COVID-19 related reports submitted to FAERS for United States (before removing cases reported before the first case of COVID-19 was confirmed). In reports where the event date is specified, we observed a median delay of 16 days (IQR 7–43 days) between report date and event date, and a mean delay of 58.57 days (95%CI 55.00–62.59 days). Since we analyze case data by “at least one dose” coverage, and effectiveness of one dose for death was found to be 72% 14–21 days after administration and 84% 21–27 days after administration (Dagan et al., 2021), we did not adjust for this estimated reporting delay.

Variant driven peaks in reported cases are not as pronounced in FAERS as they are in OWID. FAERS data are only available until 30 June 2022, thus data from the omicron wave (peak in January 2022) might be missing (Supplementary Figure S2).

#### 3.1.2 FAERS-derived COVID-19 CFR by sex and age

We queried our FAERS dataset for the known COVID-19 mortality risk factors age and male sex (Falahi and Kenarkoohi, 2022) and found that the proportion of cases with death as reported outcome was significantly higher in males (10.89%, 95%CI 10.42%–11.34%) than in females (5.02%, 95%CI 4.77%–5.27%). The same observation holds for all treatment groups individually (Supplementary Table S5). We also observed that patients with death as reported outcome were significantly older (mean age 68.11 years, 95%CI 67.46–68.72 years) than those with any other outcome (mean age 55.67 years, 95%CI 55.46–55.87 years) in the FAERS dataset and in all treatment groups (Supplementary Table S6).

Although we found a statistically significant difference in the mean age of female (56.07 years, 95%CI 55.82–56.31 years) and male (57.65 years, 95%CI 57.30–58.00 years) patients, we do not consider a difference of this magnitude and in this age range a relevant confounder.

#### 3.1.3 Age and sex distribution in FAERS pre- and post-vaccination campaign start

In the complete FAERS COVID-19 dataset, neither the difference in age (mean age 57.01 years, 95%CI 56.58–57.45 years before campaign start; mean age 56.54 years, 95%CI 56.31–56.77 years after) nor the difference in sex ratio (61.16% female, 95%CI 60.17%–62.13% before campaign start; 62.39% female, 95%CI 61.89%–62.89% after) were statistically significant (Supplementary Table S7). We thus concluded that our FAERS COVID-19 dataset does not suffer from a bias coming from changes in the age or sex distribution in reported cases over the course of the pandemic. Results for the individual treatment groups are presented in the corresponding sections.

#### 3.1.4 Vaccination effect by sex and age group on FAERS-derived COVID-19 CFRs

**FIGURE 2:**
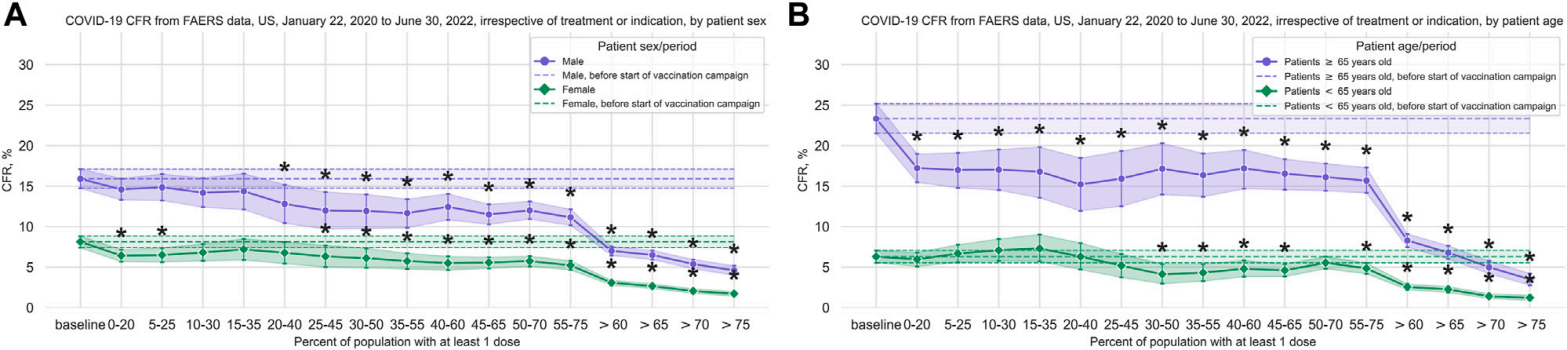
COVID-19 CFR for female and male patients, and for younger vs older patients. **(A)** CFR in percent from FAERS COVID-19 data (all reports with COVID-19 as reaction and where patient sex is specified) for the US, in 20% vaccination coverage bins, with male patients shown in blue and female patients shown in green. **(B)** CFR in percent from FAERS COVID-19 data (all reports with COVID-19 as reaction and where patient age is specified) for the United States, in 20% vaccination coverage bins, with patients ≥65 years shown in blue and patients <65 years old shown in green. Bins are indicated as (lower bound, upper bound], and the baseline period is defined as the period before the first vaccination was administered. Since a coverage of 80% had not yet been achieved at the time of data collection, we denote bins starting from 60% or higher coverage as “> *x*,” indicating that these bins include all data from coverage levels higher than x. The 95% confidence intervals are estimated using bootstrap resampling, and asterisks mark data points where the CFR is significantly different from the CFR during the baseline period (*p*-value from resampling, Benjamini-Hochberg with an accepted FDR of 5% over all bins, sexes, and age groups). Abbreviations: CFR: Case Fatality Rate; FAERS: FDA Adverse Event Reporting System.

For male patients we observed a significant reduction of the CFR after 20% of the population had received at least one dose, and for female patients we observed a significant reduction after 25% (Figure 2A; Supplementary Tables S8, S9).

For patients 65 years and older we observed a significant reduction of the CFR immediately after the start of the vaccination campaign, whereas for younger patients a reduction is only present after 30% of the population had received at least 1 dose, from 26 March 2021 on (Figure 2B; Supplementary Tables S10, S11).

### 3.2 Vaccination effect in immunotherapy groups

Note that treatment groups are monotherapy only except for the glucocorticoid group, and cases with COVID-19 as only indication are excluded (see Table 1 and Data selection in Supplementary Methods).

#### 3.2.1 Anti-CD20

**FIGURE 3:**
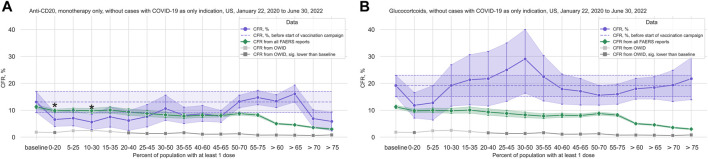
COVID-19 CFR for the anti-CD20 and the glucocorticoid treatment groups. **(A)** CFR in percent computed for all cases where only treatments from the anti-CD20 group are mentioned under Suspect Product Active Ingredient (cases with COVID-19 as only indication excluded) (shown in blue), United States only, in 20% vaccination coverage bins. **(B)** CFR for the glucocorticoid treatment group (cases with COVID-19 as only indication excluded) (shown in blue), United States only, in 20% vaccination coverage bins. Bins are indicated as (lower bound, upper bound], and the baseline period is defined as the period before the first vaccination was administered. Since a coverage of 80% had not yet been achieved at the time of data collection, we denote bins starting from 60% or higher coverage as “> *x*”, indicating that these bins include all data from coverage levels higher than x. The 95% confidence intervals are estimated using bootstrap resampling, and asterisks mark data points where the CFR in the treatment group is significantly different from the CFR during the baseline period for this group (*p*-value from resampling, Benjamini-Hochberg with an accepted FDR of 5% over all bins and treatment groups). Data for the complete FAERS COVID-19 set are shown in green (note that significant data points are not annotated for better readability; see Figure 1A for FAERS details). The grey line shows the COVID-19 CFR from OWID data, with data points where the CFR is significantly lower than during the baseline period shown in a darker grey. Abbreviations: CFR: Case Fatality Rate; FAERS: FDA Adverse Event Reporting System; OWID: Our World In Data; sig.: Significant.

In the anti-CD20 treatment group (1,907 cases, 202 deaths; median age 49 years, IQR 40–59 years; 72.0% of cases where sex is indicated concerning female patients), we observed that although there is a significant decrease in the CFR at the beginning of the vaccination campaign, the trend does not hold with increasing vaccination coverage (Figure 3A; Table 2; Supplementary Table S12). Comparing the data from before and after the vaccination campaign started, we did not find statistically significant differences in the mean age (50.52 years, 95%CI 48.69–52.25 years before campaign start; 48.70 years, 95%CI 47.75–49.64 years after campaign start) or the sex ratio in cases where sex is indicated (67.47% female, 95%CI 61.04%–72.69% before campaign start; 72.62%, 95%CI 70.16%–74.86% after) (Supplementary Table S7).

**TABLE 2:** COVID-19 CFRs from FAERS COVID-19 case reports, US only, overall and by treatment group, by vaccination coverage in the total population. Treatment groups are monotherapy only except for glucocorticoids, but within-group combinations (e.g., Ocrelizumab + Rituximab) are included. Cases with COVID-19 as only indication are excluded from the treatment group data sets. Data from 2020–01–22 to 2022–06–30, US. 95% confidence intervals (95%CI) from bootstrap resampling; CFRs that are significantly different from the baseline CFR are printed in bold (permutation test, Benjamini-Hochberg with an accepted FDR of 5% over all bins and treatment groups). Vaccination coverage bins, defined as (lower bound, upper bound], are by percentage of the total population who had received at least one dose, data from *Our World in Data* (Mathieu et al., 2020). Abbreviations: CFR: case fatality rate; COVID-19: Corona Virus Disease-2019; FAERS: FDA Adverse Event Reporting System.

Vaccination coverage in total population		CFR (95% CI) from FAERS COVID-19 reports, US, 22 January 2020 to 30 June 2022, overall and by treatment group					
**Bin, % ≥ 1 dose**	**Covered period, both dates included**	**Whole dataset**	**Anti-CD20, monotherapy**	**Anti-TNFα, monotherapy**	**Glucocorticoids, incl. combinations**	**JAK inhibitors, monotherapy**	**Thalidomide analogs, monotherapy**
*baseline*	*2020-01-22—2020-12–12*	*11.25 (10.63–11.89)*	*13.07 (9.19–16.96)*	*4.07 (2.62–5.52)*	*19.19 (15.14–22.97)*	*4.90 (3.50–6.41)*	*17.08 (14.46–19.58)*
0–20	2020-12-13—2021-03–06	**9.78 (9.11–10.44)**	**6.55 (3.99–9.12)**	5.19 (3.4–6.98)	11.83 (7.10–16.57)	7.72 (5.57–9.87)	**11.62 (9.39–13.84)**
5–25	2021-01-18—2021-03–16	**9.96 (9.14–10.81)**	7.04 (4.23–9.86)	4.57 (2.54–6.6)	12.77 (6.38–19.15)	**8.77 (6.03–11.78)**	**11.11 (8.44–13.99)**
10–30	2021-02-04—2021-03–25	**9.81 (8.87–10.77)**	**5.60 (2.80–8.40)**	6.36 (3.18–9.55)	19.23 (11.54–26.92)	5.84 (3.11–8.67)	**11.21 (7.88–14.55)**
15–35	2021-02-23—2021-04–04	10.08 (8.93–11.26)	7.53 (3.76–11.29)	3.33 (0.67–6.67)	21.33 (12.00–30.67)	6.25 (2.84–10.23)	12.56 (7.73–16.91)
20–40	2021-03-07—2021-04–13	**9.33 (8.14–10.53)**	6.17 (2.47–9.88)	4.11 (1.37–7.53)	21.74 (11.59–31.88)	5.49 (2.20–8.79)	11.25 (6.25–16.25)
25–45	2021-03-17—2021-04–27	**8.81 (7.66–10.01)**	7.64 (3.82–12.10)	3.89 (1.11–6.67)	25.00 (13.33–35.00)	6.90 (3.45–10.92)	13.92 (8.23–19.62)
30–50	2021-03-26—2021-05–18	**8.28 (7.23–9.33)**	10.62 (6.25–15.62)	1.89 (0.38–3.79)	29.09 (16.36–40.00)	8.04 (4.52–12.06)	13.12 (8.12–18.12)
35–55	2021-04-05—2021-06–28	**7.81 (6.98–8.70)**	8.11 (5.07–11.15)	1.22 (0.3–2.43)	22.47 (13.48–30.34)	6.82 (3.64–10.45)	12.62 (7.77–16.99)
40–60	2021-04-14—2021-08–13	**8.13 (7.33–8.91)**	8.68 (5.88–11.76)	**1.04 (0.26–2.07)**	17.88 (12.29–23.46)	6.79 (3.77–9.81)	13.41 (9.20–17.62)
45–65	2021-04-28—2021-10–03	**8.03 (7.42–8.67)**	7.76 (5.55–9.98)	**1.14 (0.33–1.95)**	17.09 (11.97–21.79)	5.65 (3.53–7.76)	**11.87 (9.05–14.69)**
50–70	2021-05-19—2021-11–25	**8.76 (8.18–9.34)**	13.25 (10.89–15.62)	**1.83 (0.84–2.81)**	15.58 (11.90–19.26)	5.98 (4.04–7.91)	**11.90 (9.52–14.29)**
55–75	2021-06-29—2022-01–18	**8.23 (7.72–8.74)**	14.73 (12.05–17.41)	2.80 (1.6–4.0)	16.00 (12.27–19.73)	6.73 (4.82–8.63)	**8.96 (7.30–10.63)**
>60	2021-08-14—2022-06–30	**5.02 (4.74–5.30)**	13.40 (11.01–15.78)	2.20 (1.49–2.98)	17.99 (14.14–21.85)	4.09 (3.22–5.03)	**6.15 (5.19–7.16)**
>65	2021-10-04—2022-06–30	**4.56 (4.28–4.84)**	16.09 (13.03–19.35)	2.43 (1.53–3.33)	18.46 (14.15–22.46)	3.93 (2.99–4.92)	**5.35 (4.40–6.35)**
>70	2021-11-26—2022-06–30	**3.58 (3.31–3.84)**	6.91 (3.66–10.16)	2.32 (1.33–3.32)	19.34 (13.26–24.86)	3.30 (2.38–4.30)	**4.33 (3.40–5.31)**
>75	2022-01-19—2022-06–30	**2.99 (2.72–3.26)**	5.85 (2.34–9.36)	**1.68 (0.78–2.59)**	21.74 (13.91–29.57)	**2.68 (1.79–3.66)**	**4.28 (3.19–5.37)**

#### 3.2.2 Anti-TNFα

**FIGURE 4:**
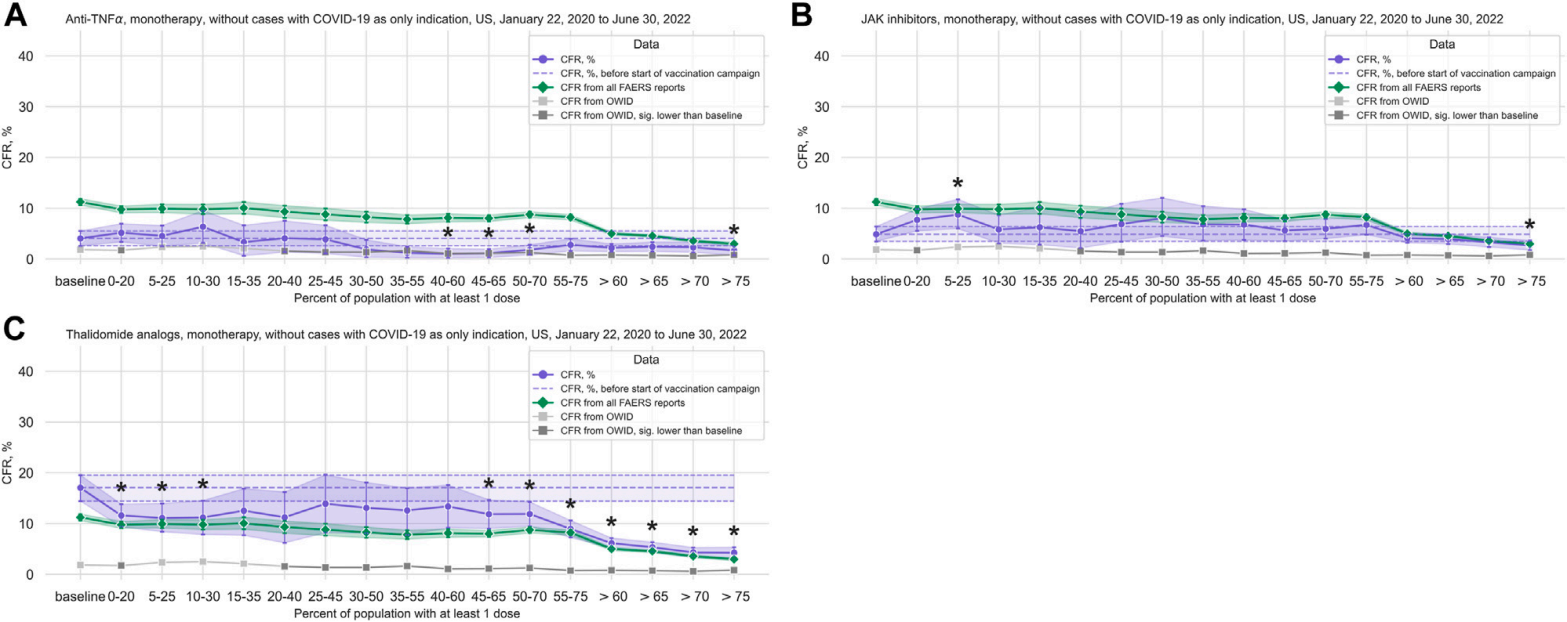
COVID-19 CFR for the anti-TNFα, JAK inhibitors, and thalidomide analogs treatment groups. **(A)** CFR for the anti-TNFα treatment group (shown in blue), United States only, in 20% vaccination coverage bins. **(B)** CFR for the JAK inhibitors treatment group (shown in blue), United States only, in 20% vaccination coverage bins. **(C)** CFR for the thalidomide analogs treatment group (shown in blue), United States only, in 20% vaccination coverage bins. All treatment groups are monotherapy only, and cases where COVID-19 is the only indication are excluded. Bins are indicated as (lower bound, upper bound], and the baseline period is defined as the period before the first vaccination was administered. Since a coverage of 80% had not yet been achieved at the time of data collection, we denote bins starting from 60% or higher coverage as “> *x*”, indicating that these bins include all data from coverage levels higher than x. The 95% confidence intervals are estimated using bootstrap resampling, and asterisks mark data points where the CFR in a treatment group is significantly different from the CFR during the baseline period for this group (*p*-value from resampling, Benjamini-Hochberg with an accepted FDR of 5% over all bins and treatment groups. Data for the complete FAERS COVID-19 set are shown in green (note that significant data points are not annotated for better readability; see Figure 1A for FAERS details). The grey line shows the COVID-19 CFR from OWID data, with data points where the CFR is significantly lower than during the baseline period shown in a darker grey. Abbreviations: CFR: Case Fatality Rate; FAERS: FDA Adverse Event Reporting System; OWID: Our World In Data; sig.: Significant.

We observed a statistically significant reduction in the CFR in the anti-TNFα treatments group (3,189 cases, 98 deaths; median age 57 years, IQR 45–66 years; 72.19% of cases where sex is indicated concerning female patients) (Figure 4A; Table 2; Supplementary Table S13). Comparing data from before and after vaccination campaign start, we did not find statistically significant differences in the mean age (54.42 years, 95%CI 53.05–55.72 years before campaign start; 54.87 years, 95%CI 53.97–55.73 years after campaign start) or the sex ratio in cases where sex is indicated (70.83% female, 95%CI 67.07%–74.14% before campaign start; 72.56%, 95%CI 70.73%–74.27% after) (Supplementary Table S7).

#### 3.2.3 Glucocorticoids

We did not see any reduction in the CFR in the glucocorticoid group (1,176 cases, 208 deaths; median age 57 years, IQR 45–68 years; 46.66% of cases where sex is indicated concerning female patients) (Figure 3B; Table 2; Supplementary Table S14). Comparing data from before and after vaccination campaign start, we found statistically significant differences in the mean age (49.34 years, 95%CI 47.03–51.63 years before campaign start; 55.86 years, 95%CI 54.44–57.21 years after campaign start) and the sex ratio in cases where sex is indicated (37.99% female, 95%CI 32.47%–43.18% before campaign start; 50.20%, 95%CI 46.49%–53.78% after) (Supplementary Table S7). While the older age of patients in the baseline period would lead to an overestimation of the decrease in CFR during the vaccination campaign, the higher prevalence of female sex after the baseline period would have the opposite effect. Although older age is associated with a higher mortality in COVID-19, the relevance of the observed age difference of less than 10 years seems clinically not relevant.

Of note, in this group not only monotherapy cases were considered (glucocorticoid only: 43 cases; combination treatments: 1,133 cases (Supplementary Table S15)). Following the hypothesis of a relevant contribution of anti-CD20 drugs to increased CFRs, we analyzed the CFR for the glucocorticoid group without cases where an anti-CD20 treatment is listed under *Suspect Product Active Ingredient* and found a slight reduction in CFR, but no significant decrease over the course of the pandemic (Supplementary Table S16; Supplementary Figure S3). Since only 44 cases were available in the 30%–50% bin for this dataset, it was excluded from the main analysis.

#### 3.2.4 JAK inhibitors

For the JAK inhibitors group (3,572 cases, 183 deaths; median age 59 years, IQR 50–65 years; 78.89% of cases where sex is indicated concerning female patients), we observed a statistically significant increase in the CFR during the first phase of the vaccination campaign with a reduction in the CFR after a coverage of 60% was achieved, similarly to what we observed in the complete FAERS COVID-19 dataset, although this reduction is only statistically significant after a coverage of 75% (Figure 4B; Table 2; Supplementary Table S17). Comparing data from before and after vaccination campaign start, we did not find statistically significant differences in the mean age (57.81 years, 95%CI 56.90–58.69 years before campaign start; 57.33 years, 95%CI 56.77–57.89 years after campaign start) or the sex ratio in cases where sex is indicated (79.46% female, 95%CI 76.36%–82.04% before campaign start; 78.70%, 95%CI 77.0%–80.27% after) (Supplementary Table S7).

#### 3.2.5 Thalidomide analogs

For the thalidomide analogs group (4,307 cases, 424 deaths; median age 68 years, IQR 61–76 years; 48.22% of cases where sex is indicated concerning female patients), we found a statistically significant decrease in CFR compared to baseline in the early stages of the vaccination campaign, and again from 45% coverage on (Figure 4C; Table 2; Supplementary Table S18). This curve displays a very similar evolution of the CFR as that of patients ≥65 years old (Figure 2B; Supplementary Table S10). Comparing the data from before and after vaccination campaign start, we did not find statistically significant differences in the mean age (67.56 years, 95%CI 66.49–68.61 years before campaign start; 67.88 years, 95%CI 67.31–68.42 years after campaign start) or the sex ratio in cases where sex is indicated (45.39% female, 95%CI 41.81%–48.75% before campaign start; 48.87%, 95%CI 47.15%–50.47% after) (Supplementary Table S7).

## 4 Discussion

The efficacy and safety of SARS-CoV-2 vaccines has been studied on population-level within the scope of the pivotal vaccination trials (Polack et al., 2020; Baden et al., 2021), and for the general population in large-scale observational studies during mass vaccination campaigns (Dagan et al., 2021). However, the efficacy of vaccinations for patients under immunotherapies has so far only been studied on individual case-level using humoral or T cell response as proxy. In addition, patients under immunotherapies were explicitly excluded from the pivotal vaccination trials (Polack et al., 2020; Baden et al., 2021). Population-level data for this vulnerable population on COVID-19 outcome with respect to vaccination coverage in the total population are still lacking. The present study aims to close this gap by analyzing COVID-19 cases reported to FAERS in combination with vaccination coverage data from OWID.

In line with a study that found that the rate ratio of COVID-19 deaths among adults ≥65 years to adults aged 18–49 years declined by 66% when comparing data from November 29—12 December 2020 to data from April 18—1 May 2021 (Christie et al., 2021), we detected a CFR decline in patients 65 years and older starting almost immediately with the start of the vaccination campaign as compared to this effect starting at 30% first-dose vaccination coverage for those under 65 years. This may serve as a proof of robustness of our approach and potentially reflects the US vaccination strategy prioritizing people 65 years and older until March—May 2021 (Centers for Disease Control and Prevention CDC, 2020; American Journal of Managed Care AJMC, 2021), leading to a higher vaccination coverage in this age group in early 2021.

We found that although the CFR decreases in the general population with increasing vaccination coverage, this effect is not observed in patients treated with CD20-targeting drugs or patients treated with glucocorticoids. This is in line with studies showing impaired humoral and cellular vaccination responses in patients under anti-CD20 treatment (Moor et al., 2021; Pri-Paz Basson et al., 2022; Wu et al., 2022) and studies showing that glucocorticoids impair the immunogenicity of the COVID-19 vaccine (Bugatti et al., 2021; Pri-Paz Basson et al., 2022). In contrast, in accordance with previous studies that found that the respective treatments do not significantly impair COVID-19 vaccination efficacy, we observed a decrease in CFRs for patients under anti-TNFα (Dimopoulou et al., 2022; Pri-Paz Basson et al., 2022; Venerito et al., 2022), JAK inhibitor (Winthrop et al., 2016; Mortezavi et al., 2021; Pri-Paz Basson et al., 2022), or thalidomide analog (Jenner et al., 2021) treatment. For the thalidomide analogs group (median patient age 68 years, IQR 61–76 years) the evolution of the CFR over the course of the vaccination campaign is similar to what we observed for patients ≥65 years old in the general population (FAERS COVID-19 dataset, irrespective of treatment). This is consistent with literature that shows a high serological response rate to COVID-19 vaccination in people treated with lenalidomide (Jenner et al., 2021) and with the earlier decrease in CFR for elderly patients (Christie et al., 2021).

Although the present study does not claim any causality between increasing vaccination coverage and reduced mortality, the observation that there is no reduction in CFR for certain at-risk groups implies that vaccination as mitigation strategy does not provide sufficient protection for those groups. Thus, our study highlights the burden of certain immunotherapies affecting the efficacy of a SARS-CoV-2 vaccination such as chronic use of glucocorticoids and anti-CD20 treatments. For these populations, other risk mitigation strategies, be it adapted vaccination regimens, passive vaccination strategies or additional non-pharmacological means on an individual- or population-based level, might be needed to significantly reduce the rate of fatal COVID-19.

Main limitations of our study based on openly available self-reported data have to be discussed.

Reported cases are not a random sample of the general population. The magnitude of the CFRs in FAERS is higher than in OWID, and we hypothesize that this might be because patients only report to FAERS if they consider the event reasonably severe, generating a possible underreporting bias for mild cases, and because patients reporting to FAERS are under treatment, which implies health issues and potentially higher mortality risk. Population characteristics vary between treatment groups, which is potentially associated with differing reporting behaviors. Reporting bias is one of the reasons why we did not draw quantitative conclusions on COVID-19 mortality risk such as comparing CFRs between datasets, countries, or treatment groups, but only analyzed relative changes in CFRs within well-defined groups.

FAERS data were comparable with OWID data in qualitative terms and several positive controls such as age and male sex demonstrated the known mortality increase. We therefore consider it appropriate to use FAERS data for exploratory qualitative analyses for hypothesis generation, since they mirror the course of the pandemic in the general population.

The results of our study do not imply a causal relation between increased vaccination coverage rate and decreased mortality. The reduction in CFR we observed for the general population could, among other reasons, be due to mitigation strategies such as mask mandates or lockdowns, improvement and better availability of treatment, lower mortality risk in more recent virus variants, or survivor bias. The absence of any reduction in CFR, on the other hand, suggests that none of the above-mentioned factors seems to reduce risk in people treated with anti-CD20 or glucocorticoids sufficiently.

Another important limitation of the present study is the reliance on self-reported data with only basic, and often incomplete, information on patients. We have no information on dosage, method, or duration of treatment administration, and information on concomitant treatments might be incomplete. Further, different reasons for use might contribute to COVID-19 severity. The most frequent reason was *Multiple Sclerosis* for anti-CD20 and *Immunosuppression* for the glucocorticoid group. However, to the best of our knowledge, no other data sources including more than 49,742 cases are available to supplement such an analysis.

The method of SARS-CoV-2 detection is not described in FAERS case reports. Therefore, cases treated as COVID-19 cases might be classified as such based on symptoms, or even suspicions, e.g., based on exposure to infected subjects. On the other hand, COVID-19 cases might not have been reported as such when other, more severe reactions were present, or when they were asymptomatic or not confirmed by testing. Due to this potential reporting bias and due to missing data on symptom severity in FAERS, conclusions regarding different phases of COVID-19 disease course (Zhou et al., 2020) cannot be drawn by our analysis.

In contrast to the other treatment groups where only monotherapy cases were considered, the glucocorticoid group mainly consisted of combination treatments. We can thus not dissect potential effects solely attributed to glucocorticoids and/or co-treatment with other immunosuppressive substances. Yet, excluding anti-CD20 co-treated patients, still no significant CFR decrease over the course of the pandemic was detected in the glucocorticoid group.

Concluding, our study provides valuable insights into the effects of the COVID-19 vaccination campaign on specific population groups as it shows that while the mortality risk in the general population is declining over the course of the pandemic, it remains high for certain at-risk patient groups despite current mitigation strategies which include vaccinations.

## Data Availability

Publicly available datasets were analyzed in this study. This data can be found here: https://www.fda.gov/drugs/questions-and-answers-fdas-adverse-event-reporting-system-faers/fda-adverse-event-reporting-system-faers-public-dashboard, https://github.com/owid/covid-19-data/tree/master/public/data.
