# Supplementary material for: The impact of immunotherapies on COVID-19 case fatality rates during the US vaccination campaign: a multidisciplinary open data analysis using FDA Adverse Event Reporting System and Our World in Data
Source: Front Pharmacol. 2023 Jun 15;14:1186404. doi: 10.3389/fphar.2023.1186404 (PMC10308012; doi:10.3389/fphar.2023.1186404)
Supplement: Supplementary file 1 [file DataSheet1.PDF]

frontiers

Supplementary Material

The impact of immunotherapies on COVID-19 Case Fatality Rates during the US vaccination campaign: A multidisciplinary open data analysis using FDA Adverse Event Reporting System and *Our World in Data*

Anke Salmen, Stefanie Marti, Andreas G. F. Hoepner, Andrew Chan, Robert Hoepner*

*** Correspondence:** Robert Hoepner: robert.hoepner@insel.ch

1 Supplementary Methods

1.1 Data selection

We downloaded all reports mentioning COVID-19 or SARS-CoV-2 as a reaction (Supplementary table 19) from FAERS (1) (downloaded on August 12, 2022) for individual COVID-19 case reports. We downloaded the complete OWID COVID-19 dataset (2) (downloaded on August 12, 2022) for population-level cases, deaths, and vaccination rates.

From our initial FAERS COVID-19 dataset (89'343 cases), we selected the countries with the most reports (US, 51'070 reports; Canada, 9'404 reports; Great Britain, 3'481 reports; Brazil, 2'215 reports; France, 1'669 reports; Italy, 1'441 reports; Spain, 1'196 reports; Germany, 1'191 reports, Colombia, 1'138 reports) and combined these data with vaccination coverage data from OWID. Cases reported to FAERS before the first confirmed case of COVID-19 in the respective country were excluded from the analysis (66'404 cases left, of which 49'742 from the US).

In a first filtering step, we listed all treatments mentioned as *Suspect Product Active Ingredient* in at least 100 reports before the start of the vaccination campaign in the respective country and in at least 100 reports after the vaccination campaign had started. For the resulting 29 active ingredients (Supplementary table 20), we manually compiled a list of active ingredients with the same mode of action to build treatment groups (Supplementary table 21).

We then compiled a set of all cases for which only treatments from one of those groups are reported under *Suspect Product Active Ingredient* (i.e., monotherapy case reports only), except for the glucocorticoid group, for which we included all cases irrespective of other treatments mentioned, since glucocorticoid long-term usage mainly occurred in different combination therapies (Supplementary table 15). We also excluded cases where only COVID-19 terms are reported under *Reason for Use* from the treatment group analyses, since e.g., glucocorticoids are recommended as treatment for severe courses of COVID-19 (hospitalized patients who require conventional oxygen (3)).

These cases were grouped into overlapping bins based on the percentage of the total population who had received at least one vaccination dose at the time of reporting to FAERS, with bins of size 20% every 5% step (see Supplementary table 2 for details on how the bins were defined). To ensure

robustness of our statistical analysis, we only considered treatment groups for which each bin contains at least 50 case reports and at least one reported death. We defined the outcome as death if death was reported in *Outcomes*, and survived otherwise. We additionally required that at least 10 deaths were reported during the baseline period (before the start of the vaccination campaign), the period from the start of the vaccination campaign until 50% of the population had received at least one dose, and the period after 50% of the population had received at least one dose.

Only the following treatment groups fulfilled the above filter criteria: anti-Cluster of Differentiation (CD) 20 (1’907 cases, 202 deaths), anti-Tumor Necrosis Factor (TNF) α (3’189 cases, 98 deaths), factor Xa inhibitors (1’267 cases, 140 deaths), glucocorticoids (1’176 cases, 208 deaths), Janus Kinase (JAK) inhibitors (3’572 cases, 183 deaths), and thalidomide analogs (4’307 cases, 424 deaths), all from the US. Factor Xa inhibitors were excluded from further analysis since the scope of the present study is immunotherapies (data presented as Supplementary figure 4 and Supplementary table 22). We used the complete FAERS COVID-19 dataset for the US (49’742 cases, 3’756 deaths) as our FAERS baseline, and the OWID data for the US as our OWID baseline (see Supplementary table 1 for OWID data).

2 Supplementary Tables

2.1 Supplementary table 1: Cohort characteristics

FAERS COVID-19 cases, US (whole dataset)	Overall	Died	Survived
**Number of reported cases**	**49742**	**3756**	**45986**
**Sex (n, %)**			
Female	28587 (57.47)	1434 (38.18)	27153 (59.05)
Male	17421 (35.02)	1897 (50.51)	15524 (33.76)
Not specified	3734 (7.51)	425 (11.32)	3309 (7.20)
**Age (Median, IQR)**	**59 (47 – 69)**	**70 (60 – 78)**	**58 (46 – 67)**
Female	58 (46 – 67)	68 (60 – 78)	57 (46 – 67)
Male	61 (48 – 70)	70 (62 – 78)	59 (46 – 69)
Not specified	62 (45 – 70)	65 (54 – 71)	60 (44 – 69)

Treatments in treatment groups	Overall	Died	Survived
**Anti-CD20, monotherapy**	**1907**	**202**	**1705**
Ocrelizumab	1142	91	1051
Ofatumumab	459	8	451
Rituximab	320	104	216
**Anti-TNFα, monotherapy**	**3189**	**98**	**3091**
Adalimumab	1807	65	1742
Certolizumab Pegol	625	12	613
Etanercept	597	11	586
Golimumab	78	1	77
Infliximab	96	9	87
**Glucocorticoids**	**1176**	**208**	**968**
Betamethasone	8	0	8
Cortisone	1	0	1
Dexamethasone	186	57	129
Dexamethasone Sodium Phosphate	7	5	2
Methylprednisolone	76	12	64
Methylprednisolone Sodium Succinate	15	9	6
Prednisolone	158	25	133
Prednisone	811	131	680
**JAK inhibitors, monotherapy**	**3572**	**183**	**3389**
Baricitinib	53	16	37
Ruxolitinib	396	64	332
Tofacitinib Citrate	2439	56	2383
Upadacitinib	686	47	639
**Thalidomide analogs, monotherapy**	**4307**	**424**	**1835**
Lenalidomide	3350	337	3013
Pomalidomide	934	80	854
Thalidomide	54	13	41

Indications (top 5 *Reason for Use*)	Overall	Died	Survived
**Whole dataset**			
Product Used For Unknown Indication	7216	548	6668
Covid-19 Treatment	3662	17	3645
No reason specified	3212	191	3021
Rheumatoid Arthritis	2949	125	2824
Plasma Cell Myeloma	2530	272	2258
**Anti-CD20, monotherapy**			
Multiple Sclerosis	391	24	367
Relapsing Multiple Sclerosis	272	19	253
Product Used For Unknown Indication	234	39	195
Relapsing-Remitting Multiple Sclerosis	194	16	178s
Rheumatoid Arthritis	106	29	77
**Anti-TNFα, monotherapy**			
Rheumatoid Arthritis	897	27	870
Product Used For Unknown Indication	262	20	242
Psoriasis	217	9	208
Psoriatic Arthropathy	205	5	200
Crohn's Disease	190	6	184
**Glucocorticoids**			
Immunosuppression	127	18	109
Immunosuppressant Drug Therapy	111	19	92
Systemic Lupus Erythematosus	60	0	60
Prophylaxis Against Transplant Rejection	48	10	38
Plasma Cell Myeloma	44	23	21
**JAK inhibitors, monotherapy**			
Rheumatoid Arthritis	1345	50	1295
No reason specified	1145	34	1111
Covid-19 Immunisation;Rheumatoid Arthritis	123	3	120
Psoriatic Arthropathy	92	2	90
Colitis Ulcerative	66	1	65
**Thalidomide analogs, monotherapy**			
Plasma Cell Myeloma	2352	221	2131
Plasma Cell Myeloma;Product Used For Unknown Indication	1093	92	1001
Product Used For Unknown Indication	96	9	87
Myelodysplastic Syndrome	39	6	33
Follicular Lymphoma	36	4	32

Reporter			
**Whole dataset**			
Consumer	26471	1382	25089
Healthcare Professional	22774	2321	20453
Not Specified	497	53	444
**Anti-CD20, monotherapy**			
Consumer	1152	50	1102
Healthcare Professional	736	145	591
Not Specified	19	7	12

	Overall	Died	Survived
**Anti-TNFα, monotherapy**			
Consumer	2012	70	1942
Healthcare Professional	1146	28	1118
Not Specified	31	0	31
**Glucocorticoids**			
Consumer	90	7	83
Healthcare Professional	1071	199	872
Not Specified	15	2	13
**JAK inhibitors, monotherapy**			
Consumer	1568	97	1471
Healthcare Professional	1988	86	1902
Not Specified	16	0	16
**Thalidomide analogs, monotherapy**			
Consumer	374	31	343
Healthcare Professional	3929	392	3537
Not Specified	4	1	3

Supplementary table 1: Cohort characteristics. Basic characteristics of the whole FAERS COVID-19 dataset for US, and case and death counts for the individual treatment groups, overall and by treatment, by top reasons for use, and by reporter type. COVID-19 cases are all cases reported to FAERS with a COVID-19 related term in *Reactions*. Treatment groups are monotherapy only except for glucocorticoids, but within-group combinations (e.g. Ocrelizumab + Rituximab) are included. Cases with COVID-19 as only indication under *Reason for Use* are excluded from the treatment group data sets. Data from 2020-01-22 to 2022-06-30, US. Cases are counted as died if death was reported in *Outcomes*, and as survived otherwise. Note that since within-group combinations are allowed, the sum over all cases for all individual treatments within a treatment group may be greater than the total number of cases for the entire treatment group. ***Abbreviations:*** IQR: Interquartile range, the 25th and 75th percentile; COVID-19: Corona Virus Disease-2019; FAERS: FDA Adverse Event Reporting System.

2.2 Supplementary table 2: Bins for the analysis of CFR by vaccination coverage

Bin, % of population	Covered period (both dates included)	n cases (n deaths, % deaths) in OWID
baseline	2020-01-22 – 2020-12-12	16220548 (300092, 1.85%)
0 – 20	2020-12-13 – 2021-03-06	12904350 (222418, 1.72%)
5 – 25	2021-01-18 – 2021-03-16	5526108 (131079, 2.37%)
10 – 30	2021-02-04 – 2021-03-25	3474292 (87846, 2.53%)
15 – 35	2021-02-23 – 2021-04-04	2493654 (52397, 2.10%)
20 – 40	2021-03-07 – 2021-04-13	2326777 (36978, 1.59%)
25 – 45	2021-03-17 – 2021-04-27	2633838 (35709, 1.36%)
30 – 50	2021-03-26 – 2021-05-18	2926343 (39644, 1.35%)
35 – 55	2021-04-05 – 2021-06-28	2937097 (48725, 1.66%)
40 – 60	2021-04-14 – 2021-08-13	5268205 (57945, 1.10%)
45 – 65	2021-04-28 – 2021-10-03	11495446 (130652, 1.14%)
50 – 70	2021-05-19 – 2021-11-25	15052996 (191314, 1.27%)
55 – 75	2021-06-29 – 2022-01-18	34120142 (256191, 0.75%)
> 60	2021-08-14 – 2022-06-30	50955831 (401143, 0.79%)
> 65	2021-10-04 – 2022-06-30	43896977 (318568, 0.73%)
> 70	2021-11-26 – 2022-06-30	39513725 (244334, 0.62%)
> 75	2022-01-19 – 2022-06-30	19798328 (162331, 0.82%)

Supplementary table 2: Vaccination coverage bins for the US. This table lists the bins we used for our analysis of the CFR by vaccination coverage, defined as (lower bound, upper bound]. Vaccination coverage data is from OWID. Case and death counts for FAERS and treatment groups are listed in the respective result tables. ***Abbreviations:*** OWID: Our World In Data.

2.3 Supplementary table 3: CFR by vaccination coverage in OWID, US

Bin, % 1 dose	Covered period	n cases (n deaths)	CFR (95% CI)	p vs. baseline
baseline	2020-01-22 – 2020-12-12	16220548 (300092)	1.85 (1.84 – 1.86)	-
0 – 20	2020-12-13 – 2021-03-06	12904350 (222418)	1.72 (1.72 – 1.73)	**< 0.001**
5 – 25	2021-01-18 – 2021-03-16	5526108 (131079)	2.37 (2.36 – 2.38)	**< 0.001**
10 – 30	2021-02-04 – 2021-03-25	3474292 (87846)	2.53 (2.51 – 2.55)	**< 0.001**
15 – 35	2021-02-23 – 2021-04-04	2493654 (52397)	2.10 (2.08 – 2.12)	**< 0.001**
20 – 40	2021-03-07 – 2021-04-13	2326777 (36978)	1.59 (1.57 – 1.61)	**< 0.001**
25 – 45	2021-03-17 – 2021-04-27	2633838 (35709)	1.36 (1.34 – 1.37)	**< 0.001**
30 – 50	2021-03-26 – 2021-05-18	2926343 (39644)	1.35 (1.34 – 1.37)	**< 0.001**
35 – 55	2021-04-05 – 2021-06-28	2937097 (48725)	1.66 (1.64 – 1.67)	**< 0.001**
40 – 60	2021-04-14 – 2021-08-13	5268205 (57945)	1.10 (1.09 – 1.11)	**< 0.001**
45 – 65	2021-04-28 – 2021-10-03	11495446 (130652)	1.14 (1.13 – 1.14)	**< 0.001**
50 – 70	2021-05-19 – 2021-11-25	15052996 (191314)	1.27 (1.27 – 1.28)	**< 0.001**
55 – 75	2021-06-29 – 2022-01-18	34120142 (256191)	0.75 (0.75 – 0.75)	**< 0.001**
> 60	2021-08-14 – 2022-06-30	50955831 (401143)	0.79 (0.78 – 0.79)	**< 0.001**
> 65	2021-10-04 – 2022-06-30	43896977 (318568)	0.73 (0.72 – 0.73)	**< 0.001**
> 70	2021-11-26 – 2022-06-30	39513725 (244334)	0.62 (0.62 – 0.62)	**< 0.001**
> 75	2022-01-19 – 2022-06-30	19798328 (162331)	0.82 (0.82 – 0.82)	**< 0.001**

Supplementary table 3: CFR from OWID data, US. CFR in percent, computed for OWID data, US only. The indicated bins are defined as (lower bound, upper bound], the 95% confidence intervals for the CFR are the Wilson score interval, the p-value is computed using the G-test, and we employ the Benjamini-Hochberg procedure with an accepted FDR of 5% over all bins and treatment groups to account for multiple testing. ***Abbreviations:*** CFR: Case Fatality Rate, OWID: Our World In Data.

2.4 Supplementary table 4: CFR by vaccination coverage in FAERS COVID-19 data

Bin, % 1 dose	Covered period	n cases (n deaths)	CFR (95% CI)	p vs. baseline
baseline	2020-01-22 – 2020-12-12	10424 (1173)	11.25 (10.63 – 11.89)	-
0 – 20	2020-12-13 – 2021-03-06	7708 (754)	9.78 (9.11 – 10.44)	**0.002**
5 – 25	2021-01-18 – 2021-03-16	5173 (515)	9.96 (9.14 – 10.81)	**0.014**
10 – 30	2021-02-04 – 2021-03-25	3834 (376)	9.81 (8.87 – 10.77)	**0.015**
15 – 35	2021-02-23 – 2021-04-04	2531 (255)	10.08 (8.93 – 11.26)	0.094
20 – 40	2021-03-07 – 2021-04-13	2175 (203)	9.33 (8.14 – 10.53)	**0.007**
25 – 45	2021-03-17 – 2021-04-27	2258 (199)	8.81 (7.66 – 10.01)	**0.001**
30 – 50	2021-03-26 – 2021-05-18	2668 (221)	8.28 (7.23 – 9.33)	**< 0.001**
35 – 55	2021-04-05 – 2021-06-28	3724 (291)	7.81 (6.98 – 8.70)	**< 0.001**
40 – 60	2021-04-14 – 2021-08-13	4799 (390)	8.13 (7.33 – 8.91)	**< 0.001**
45 – 65	2021-04-28 – 2021-10-03	7455 (599)	8.03 (7.42 – 8.67)	**< 0.001**
50 – 70	2021-05-19 – 2021-11-25	9619 (843)	8.76 (8.18 – 9.34)	**< 0.001**
55 – 75	2021-06-29 – 2022-01-18	11379 (937)	8.23 (7.72 – 8.74)	**< 0.001**
> 60	2021-08-14 – 2022-06-30	24636 (1236)	5.02 (4.74 – 5.30)	**< 0.001**
> 65	2021-10-04 – 2022-06-30	21290 (971)	4.56 (4.28 – 4.84)	**< 0.001**
> 70	2021-11-26 – 2022-06-30	18137 (649)	3.58 (3.31 – 3.84)	**< 0.001**
> 75	2022-01-19 – 2022-06-30	14874 (444)	2.99 (2.72 – 3.26)	**< 0.001**

Supplementary table 4: CFR for the complete FAERS COVID-19 US dataset. CFR in percent, computed for the FAERS COVID-19 dataset, all patients irrespective of treatment or indication. The indicated bins are defined as (lower bound, upper bound], the 95% confidence intervals for the CFR are estimated using a bootstrapping approach, the p-value is computed using a resampling procedure, and we employ the Benjamini-Hochberg procedure with an accepted FDR of 5% over all bins and treatment groups to account for multiple testing. ***Abbreviations:*** CFR: Case Fatality Rate; COVID-19: Corona Virus Disease-2019; FAERS: FDA Adverse Event Reporting System.

2.5 Supplementary table 5: Sex as risk factor in FAERS COVID-19 data, US

Dataset	Female % died (95% CI)	Male % died (95% CI)	p
Whole dataset	5.02 (4.77 – 5.27)	10.89 (10.42 – 11.34)	**< 0.001**
Anti-CD20	6.48 (5.12 – 7.93)	12.61 (9.57 – 15.65)	**< 0.001**
Anti-TNFα	2.08 (1.54 – 2.71)	5.39 (3.99 – 6.92)	**< 0.001**
Glucocorticoids	11.29 (8.47 – 14.11)	22.05 (18.69 – 25.40)	**< 0.001**
JAK inhibitors	2.70 (2.10 – 3.35)	7.69 (5.73 – 9.80)	**< 0.001**
Thalidomide analogs	8.69 (7.48 – 9.89)	10.88 (9.62 – 12.22)	**0.020**

Supplementary table 5: Sex as risk factor, by treatment group in FAERS COVID-19 cases where sex is specified. This table shows the fraction of reports with death as reported outcome for female and for male patients. The 95% confidence intervals were estimated using bootstrapping, and the p-values for the difference were estimated using a resampling procedure (9’999 resamples). Significant differences (Benjamini-Hochberg, accepted FDR 5% over all comparisons for age and sex) are highlighted in bold.

2.6 Supplementary table 6: Age as risk factor in FAERS COVID-19 data, US

Dataset	Died, age (years; mean, 95%CI)	Survived, age (years; mean, 95%CI)	p
Whole dataset	68.11 (67.46 – 68.72)	55.67 (55.46 – 55.87)	**< 0.001**
Anti-CD20	55.64 (52.68 – 58.64)	48.40 (47.53 – 49.27)	**< 0.001**
Anti-TNFα	67.15 (63.78 – 70.71)	54.42 (53.65 – 55.15)	**< 0.001**
Glucocorticoids	64.34 (62.06 – 66.26)	51.32 (49.92 – 52.62)	**< 0.001**
JAK inhibitors	67.83 (65.59 – 70.00)	57.10 (56.62 – 57.57)	**< 0.001**
Thalidomide analogs	72.49 (71.18 – 73.73)	67.17 (66.63 – 67.68)	**< 0.001**

Supplementary table 6: Age as risk factor, by treatment group in FAERS COVID-19 cases where patient age is specified. This table shows the mean age of the patients with death as reported outcome and of all other patients. The 95% confidence intervals were estimated using bootstrapping, and the p-values for the difference were estimated using a resampling procedure (9’999 resamples). Significant differences (Benjamini-Hochberg, accepted FDR 5% over all comparisons for age and sex) are highlighted in bold.

2.7 Supplementary table 7: Age and sex distribution before and after campaign start

Variable	Dataset	Baseline	Campaign	p
Age (years; mean, 95% CI)	Whole dataset	57.01 (56.58 – 57.45)	56.54 (56.31 – 56.77)	0.064
	Anti-CD20	50.52 (48.69 – 52.25)	48.70 (47.75 – 49.64)	0.095
	Anti-TNFα	54.42 (53.05 – 55.72)	54.87 (53.97 – 55.73)	0.592
	Glucocorticoids	49.34 (47.03 – 51.63)	55.86 (54.44 – 57.21)	**< 0.001**
	Glucocorticoids (no anti-CD20)	49.99 (47.62 – 52.28)	56.48 (55.04 – 57.89)	**< 0.001**
	JAK inhibitors	57.81 (56.90 – 58.69)	57.33 (56.77 – 57.89)	0.380
	Thalidomide analogs	67.56 (66.49 – 68.61)	67.88 (67.31 – 68.42)	0.616
% female (95% CI)	Whole dataset	61.16 (60.17 – 62.13)	62.39 (61.89 – 62.89)	0.032
	Anti-CD20	67.47 (61.04 – 72.69)	72.62 (70.16 – 74.86)	0.115
	Anti-TNFα	70.83 (67.07 – 74.14)	72.56 (70.73 – 74.27)	0.410
	Glucocorticoids	37.99 (32.47 – 43.18)	50.20 (46.49 – 53.77)	**< 0.001**
	Glucocorticoids (no anti-CD20)	36.73 (30.95 – 42.18)	51.26 (47.29 – 54.96)	**< 0.001**
	JAK inhibitors	79.46 (76.36 – 82.04)	78.71 (76.98 – 80.27)	0.700
	Thalidomide analogs	45.39 (41.81 – 48.75)	48.87 (47.15 – 50.47)	0.083

Supplementary table 7: Age and sex distribution in FAERS COVID-19 cases before and after vaccination campaign start, per treatment group. This table shows the mean age for each group during the baseline period and during the vaccination campaign period. The 95% confidence intervals were estimated using bootstrapping, and the p-values for the difference were estimated using a resampling procedure (9’999 resamples). Significant differences (Benjamini-Hochberg, accepted FDR 5% over all comparisons) are highlighted in bold.

2.8 Supplementary table 8: CFR by vaccination coverage in female patients

Bin, % 1 dose	Covered period	n cases (n deaths)	CFR (95% CI)	p vs. baseline
baseline	2020-01-22 – 2020-12-12	5799 (471)	8.12 (7.40 – 8.83)	-
0 – 20	2020-12-13 – 2021-03-06	4379 (281)	6.42 (5.66 – 7.15)	**0.002**
5 – 25	2021-01-18 – 2021-03-16	3011 (195)	6.48 (5.61 – 7.37)	**0.006**
10 – 30	2021-02-04 – 2021-03-25	2236 (152)	6.80 (5.77 – 7.87)	0.049
15 – 35	2021-02-23 – 2021-04-04	1460 (105)	7.19 (5.89 – 8.49)	0.259
20 – 40	2021-03-07 – 2021-04-13	1273 (86)	6.76 (5.42 – 8.09)	0.109
25 – 45	2021-03-17 – 2021-04-27	1315 (83)	6.31 (5.02 – 7.68)	**0.026**
30 – 50	2021-03-26 – 2021-05-18	1542 (94)	6.10 (4.93 – 7.33)	**0.007**
35 – 55	2021-04-05 – 2021-06-28	2180 (125)	5.73 (4.77 – 6.70)	**0.001**
40 – 60	2021-04-14 – 2021-08-13	2801 (154)	5.50 (4.64 – 6.32)	**< 0.001**
45 – 65	2021-04-28 – 2021-10-03	4337 (241)	5.56 (4.87 – 6.23)	**< 0.001**
50 – 70	2021-05-19 – 2021-11-25	5613 (322)	5.74 (5.11 – 6.34)	**< 0.001**
55 – 75	2021-06-29 – 2022-01-18	6578 (343)	5.21 (4.67 – 5.76)	**< 0.001**
> 60	2021-08-14 – 2022-06-29	14335 (442)	3.08 (2.80 – 3.37)	**< 0.001**
> 65	2021-10-04 – 2022-06-29	12398 (329)	2.65 (2.37 – 2.94)	**< 0.001**
> 70	2021-11-26 – 2022-06-29	10562 (216)	2.05 (1.78 – 2.32)	**< 0.001**
> 75	2022-01-19 – 2022-06-29	8707 (149)	1.71 (1.44 – 1.99)	**< 0.001**

Supplementary table 8: CFR for the FAERS COVID-19 US dataset, female patients. COVID-19 CFR in percent from all COVID-19 FAERS reports, irrespective of treatment or indication, where patient sex is indicated, female patients only. The 95% confidence intervals for the CFR are estimated using a bootstrapping approach, the p-value is computed using a resampling procedure, and we employ the Benjamini-Hochberg procedure with an accepted FDR of 5% over all bins, sexes, and age groups to account for multiple testing. ***Abbreviations:*** CFR: Case Fatality Rate.

2.9 Supplementary table 9: CFR by vaccination coverage in male patients

Bin, % 1 dose	Covered period	n cases (n deaths)	CFR (95% CI)	p vs. baseline
baseline	2020-01-22 – 2020-12-12	3683 (586)	15.91 (14.74 – 17.11)	-
0 – 20	2020-12-13 – 2021-03-06	2856 (417)	14.60 (13.31 – 15.93)	0.150
5 – 25	2021-01-18 – 2021-03-16	1850 (275)	14.86 (13.24 – 16.49)	0.336
10 – 30	2021-02-04 – 2021-03-25	1387 (197)	14.20 (12.40 – 16.01)	0.144
15 – 35	2021-02-23 – 2021-04-04	925 (133)	14.38 (12.11 – 16.54)	0.270
20 – 40	2021-03-07 – 2021-04-13	757 (97)	12.81 (10.44 – 15.19)	**0.033**
25 – 45	2021-03-17 – 2021-04-27	784 (94)	11.99 (9.69 – 14.29)	**0.005**
30 – 50	2021-03-26 – 2021-05-18	872 (104)	11.93 (9.75 – 13.99)	**0.003**
35 – 55	2021-04-05 – 2021-06-28	1208 (141)	11.67 (9.85 – 13.41)	**0.001**
40 – 60	2021-04-14 – 2021-08-13	1614 (201)	12.45 (10.84 – 14.06)	**0.002**
45 – 65	2021-04-28 – 2021-10-03	2564 (295)	11.51 (10.30 – 12.75)	**< 0.001**
50 – 70	2021-05-19 – 2021-11-25	3275 (393)	12.00 (10.93 – 13.13)	**< 0.001**
55 – 75	2021-06-29 – 2022-01-18	3915 (436)	11.14 (10.17 – 12.13)	**< 0.001**
> 60	2021-08-14 – 2022-06-29	8511 (596)	7.00 (6.44 – 7.54)	**< 0.001**
> 65	2021-10-04 – 2022-06-29	7333 (476)	6.49 (5.93 – 7.06)	**< 0.001**
> 70	2021-11-26 – 2022-06-29	6322 (340)	5.38 (4.81 – 5.93)	**< 0.001**
> 75	2022-01-19 – 2022-06-29	5174 (238)	4.60 (4.02 – 5.16)	**< 0.001**

Supplementary table 9: CFR for the FAERS COVID-19 US dataset, male patients. COVID-19 CFR in percent from all COVID-19 FAERS reports, irrespective of treatment or indication, where patient sex is indicated, male patients only. The 95% confidence intervals for the CFR are estimated using a bootstrapping approach, the p-value is computed using a resampling procedure, and we employ the Benjamini-Hochberg procedure with an accepted FDR of 5% over all bins, sexes, and age groups to account for multiple testing. ***Abbreviations:*** CFR: Case Fatality Rate.

2.10 Supplementary table 10: CFR by vaccination coverage in patients 65 years and older

Bin, % 1 dose	Covered period	n cases (n deaths)	CFR (95% CI)	p vs. baseline
baseline	2020-01-22 – 2020-12-12	2061 (481)	23.34 (21.54 – 25.18)	-
0 – 20	2020-12-13 – 2021-03-06	1787 (308)	17.24 (15.50 – 18.97)	**< 0.001**
5 – 25	2021-01-18 – 2021-03-16	1136 (193)	16.99 (14.79 – 19.10)	**< 0.001**
10 – 30	2021-02-04 – 2021-03-25	840 (143)	17.02 (14.52 – 19.52)	**< 0.001**
15 – 35	2021-02-23 – 2021-04-04	560 (94)	16.79 (13.57 – 19.82)	**0.002**
20 – 40	2021-03-07 – 2021-04-13	460 (70)	15.22 (11.96 – 18.48)	**< 0.001**
25 – 45	2021-03-17 – 2021-04-27	471 (75)	15.92 (12.53 – 19.32)	**0.001**
30 – 50	2021-03-26 – 2021-05-18	537 (92)	17.13 (13.97 – 20.30)	**0.003**
35 – 55	2021-04-05 – 2021-06-28	715 (117)	16.36 (13.71 – 19.02)	**< 0.001**
40 – 60	2021-04-14 – 2021-08-13	960 (165)	17.19 (14.69 – 19.48)	**0.001**
45 – 65	2021-04-28 – 2021-10-03	1495 (247)	16.52 (14.58 – 18.33)	**< 0.001**
50 – 70	2021-05-19 – 2021-11-25	1856 (299)	16.11 (14.44 – 17.78)	**< 0.001**
55 – 75	2021-06-29 – 2022-01-18	2060 (323)	15.68 (14.17 – 17.28)	**< 0.001**
> 60	2021-08-14 – 2022-06-29	4188 (347)	8.29 (7.45 – 9.10)	**< 0.001**
> 65	2021-10-04 – 2022-06-29	3522 (239)	6.79 (5.99 – 7.64)	**< 0.001**
> 70	2021-11-26 – 2022-06-29	2964 (148)	4.99 (4.22 – 5.77)	**< 0.001**
> 75	2022-01-19 – 2022-06-29	2487 (87)	3.50 (2.77 – 4.22)	**< 0.001**

Supplementary table 10: CFR for the FAERS COVID-19 US dataset, patients 65 years old and older. COVID-19 CFR in percent from FAERS, irrespective of treatment or indication, for all reports where patient age is indicated, if patient age ≥ 65 years. The 95% confidence intervals for the CFR are estimated using a bootstrapping approach, the p-value is computed using a resampling procedure, and we employ the Benjamini-Hochberg procedure with an accepted FDR of 5% over all bins and age groups to account for multiple testing. ***Abbreviations:*** CFR: Case Fatality Rate.

2.11 Supplementary table 11: CFR by vaccination coverage in patients under 65 years

Bin, % 1 dose	Covered period	n cases (n deaths)	CFR (95% CI)	p vs. baseline
baseline	2020-01-22 – 2020-12-12	3837 (241)	6.28 (5.53 – 7.06)	-
0 – 20	2020-12-13 – 2021-03-06	2827 (168)	5.94 (5.09 – 6.81)	0.603
5 – 25	2021-01-18 – 2021-03-16	1935 (129)	6.67 (5.58 – 7.75)	0.615
10 – 30	2021-02-04 – 2021-03-25	1426 (101)	7.08 (5.75 – 8.49)	0.317
15 – 35	2021-02-23 – 2021-04-04	931 (68)	7.30 (5.69 – 9.02)	0.278
20 – 40	2021-03-07 – 2021-04-13	827 (52)	6.29 (4.72 – 7.98)	> 0.9995
25 – 45	2021-03-17 – 2021-04-27	908 (47)	5.18 (3.74 – 6.61)	0.234
30 – 50	2021-03-26 – 2021-05-18	1043 (43)	4.12 (2.97 – 5.37)	**0.010**
35 – 55	2021-04-05 – 2021-06-28	1434 (62)	4.32 (3.28 – 5.37)	**0.006**
40 – 60	2021-04-14 – 2021-08-13	1856 (89)	4.80 (3.83 – 5.82)	**0.024**
45 – 65	2021-04-28 – 2021-10-03	2911 (134)	4.60 (3.85 – 5.36)	**0.003**
50 – 70	2021-05-19 – 2021-11-25	3547 (196)	5.53 (4.82 – 6.29)	0.183
55 – 75	2021-06-29 – 2022-01-18	3919 (190)	4.85 (4.18 – 5.51)	**0.009**
> 60	2021-08-14 – 2022-06-29	8381 (215)	2.57 (2.23 – 2.91)	**< 0.001**
> 65	2021-10-04 – 2022-06-29	7038 (158)	2.24 (1.89 – 2.60)	**< 0.001**
> 70	2021-11-26 – 2022-06-29	6040 (85)	1.41 (1.13 – 1.71)	**< 0.001**
> 75	2022-01-19 – 2022-06-29	5095 (62)	1.22 (0.92 – 1.53)	**< 0.001**

Supplementary table 11: CFR for the FAERS COVID-19 US dataset, patients under 65 years. COVID-19 CFR in percent from FAERS, irrespective of treatment or indication, for all reports where patient age is indicated, if patient age < 65 years. The 95% confidence intervals for the CFR are estimated using a bootstrapping approach, the p-value is computed using a resampling procedure, and we employ the Benjamini-Hochberg procedure with an accepted FDR of 5% over all bins and age groups to account for multiple testing. ***Abbreviations:*** CFR: Case Fatality Rate.

2.12 Supplementary table 12: CFR by vaccination coverage for the anti-CD20 treatment group

Bin, % 1 dose	Covered period	n cases (n deaths)	CFR (95% CI)	p vs. baseline
baseline	2020-01-22 – 2020-12-12	283 (37)	13.07 (9.19 – 16.96)	–
0 – 20	2020-12-13 – 2021-03-06	351 (23)	6.55 (3.99 – 9.12)	**0.009**
5 – 25	2021-01-18 – 2021-03-16	284 (20)	7.04 (4.23 – 9.86)	0.019
10 – 30	2021-02-04 – 2021-03-25	250 (14)	5.60 (2.80 – 8.40)	**0.005**
15 – 35	2021-02-23 – 2021-04-04	186 (14)	7.53 (3.76 – 11.29)	0.075
20 – 40	2021-03-07 – 2021-04-13	162 (10)	6.17 (2.47 – 9.88)	0.034
25 – 45	2021-03-17 – 2021-04-27	157 (12)	7.64 (3.82 – 12.10)	0.118
30 – 50	2021-03-26 – 2021-05-18	160 (17)	10.62 (6.25 – 15.62)	0.551
35 – 55	2021-04-05 – 2021-06-28	296 (24)	8.11 (5.07 – 11.15)	0.070
40 – 60	2021-04-14 – 2021-08-13	357 (31)	8.68 (5.88 – 11.76)	0.092
45 – 65	2021-04-28 – 2021-10-03	541 (42)	7.76 (5.55 – 9.98)	0.022
50 – 70	2021-05-19 – 2021-11-25	762 (101)	13.25 (10.89 – 15.62)	> 0.9995
55 – 75	2021-06-29 – 2022-01-18	672 (99)	14.73 (12.05 – 17.41)	0.572
> 60	2021-08-14 – 2022-06-30	754 (101)	13.40 (11.01 – 15.78)	0.991
> 65	2021-10-04 – 2022-06-30	522 (84)	16.09 (13.03 – 19.35)	0.295
> 70	2021-11-26 – 2022-06-30	246 (17)	6.91 (3.66 – 10.16)	0.026
> 75	2022-01-19 – 2022-06-30	171 (10)	5.85 (2.34 – 9.36)	0.022

Supplementary table 12: CFR for the anti-CD20 treatment group. CFR in percent for the anti-CD20 treatment group (only treatments from the anti-CD20 group mentioned under *Suspect Product Active Ingredient*; cases where COVID-19 is the only indication excluded). The 95%CI for the CFR are estimated using a bootstrapping approach, the p-value is computed using a resampling procedure, and we employ the Benjamini-Hochberg procedure with an accepted FDR of 5% over all bins and treatment groups to account for multiple testing. ***Abbreviations:*** CFR: Case Fatality Rate.

2.13 Supplementary table 13: CFR by vaccination coverage for the anti-TNFα treatment group

Bin, % 1 dose	Covered period	n cases (n deaths)	CFR (95% CI)	p vs. baseline
baseline	2020-01-22 – 2020-12-12	688 (28)	4.07 (2.62 – 5.52)	-
0 – 20	2020-12-13 – 2021-03-06	559 (29)	5.19 (3.4 – 6.98)	0.420
5 – 25	2021-01-18 – 2021-03-16	394 (18)	4.57 (2.54 – 6.6)	0.803
10 – 30	2021-02-04 – 2021-03-25	220 (14)	6.36 (3.18 – 9.55)	0.235
15 – 35	2021-02-23 – 2021-04-04	150 (5)	3.33 (0.67 – 6.67)	0.901
20 – 40	2021-03-07 – 2021-04-13	146 (6)	4.11 (1.37 – 7.53)	> 0.9995
25 – 45	2021-03-17 – 2021-04-27	180 (7)	3.89 (1.11 – 6.67)	> 0.9995
30 – 50	2021-03-26 – 2021-05-18	264 (5)	1.89 (0.38 – 3.79)	0.135
35 – 55	2021-04-05 – 2021-06-28	329 (4)	1.22 (0.3 – 2.43)	0.018
40 – 60	2021-04-14 – 2021-08-13	386 (4)	1.04 (0.26 – 2.07)	**0.005**
45 – 65	2021-04-28 – 2021-10-03	614 (7)	1.14 (0.33 – 1.95)	**0.001**
50 – 70	2021-05-19 – 2021-11-25	711 (13)	1.83 (0.84 – 2.81)	**0.016**
55 – 75	2021-06-29 – 2022-01-18	750 (21)	2.80 (1.6 – 4.0)	0.243
> 60	2021-08-14 – 2022-06-30	1410 (31)	2.20 (1.49 – 2.98)	0.026
> 65	2021-10-04 – 2022-06-30	1112 (27)	2.43 (1.53 – 3.33)	0.072
> 70	2021-11-26 – 2022-06-30	904 (21)	2.32 (1.33 – 3.32)	0.060
> 75	2022-01-19 – 2022-06-30	773 (13)	1.68 (0.78 – 2.59)	**0.010**

Supplementary table 13: CFR for the anti-TNFα treatment group. CFR in percent for the anti-TNFα treatment group (only treatments from the anti-TNFα group mentioned under *Suspect Product Active Ingredient*; cases where COVID-19 is the only indication excluded). The 95%CI for the CFR are estimated using a bootstrapping approach, the p-value is computed using a resampling procedure, and we employ the Benjamini-Hochberg procedure with an accepted FDR of 5% over all bins and treatment groups to account for multiple testing. ***Abbreviations:*** CFR: Case Fatality Rate.

2.14 Supplementary table 14: CFR by vaccination coverage for the glucocorticoid treatment group

Bin, % 1 dose	Covered period	n cases (n deaths)	CFR (95% CI)	p vs. baseline
baseline	2020-01-22 – 2020-12-12	370 (71)	19.19 (15.14 – 22.97)	–
0 – 20	2020-12-13 – 2021-03-06	169 (20)	11.83 (7.10 – 16.57)	0.038
5 – 25	2021-01-18 – 2021-03-16	94 (12)	12.77 (6.38 – 19.15)	0.204
10 – 30	2021-02-04 – 2021-03-25	104 (20)	19.23 (11.54 – 26.92)	> 0.9995
15 – 35	2021-02-23 – 2021-04-04	75 (16)	21.33 (12.00 – 30.67)	0.784
20 – 40	2021-03-07 – 2021-04-13	69 (15)	21.74 (11.59 – 31.88)	0.717
25 – 45	2021-03-17 – 2021-04-27	60 (15)	25.00 (13.33 – 35.00)	0.372
30 – 50	2021-03-26 – 2021-05-18	55 (16)	29.09 (16.36 – 40.00)	0.133
35 – 55	2021-04-05 – 2021-06-28	89 (20)	22.47 (13.48 – 30.34)	0.579
40 – 60	2021-04-14 – 2021-08-13	179 (32)	17.88 (12.29 – 23.46)	0.822
45 – 65	2021-04-28 – 2021-10-03	234 (40)	17.09 (11.97 – 21.79)	0.601
50 – 70	2021-05-19 – 2021-11-25	353 (55)	15.58 (11.90 – 19.26)	0.240
55 – 75	2021-06-29 – 2022-01-18	375 (60)	16.00 (12.27 – 19.73)	0.300
> 60	2021-08-14 – 2022-06-30	389 (70)	17.99 (14.14 – 21.85)	0.759
> 65	2021-10-04 – 2022-06-30	325 (60)	18.46 (14.15 – 22.46)	0.899
> 70	2021-11-26 – 2022-06-30	181 (35)	19.34 (13.26 – 24.86)	> 0.9995
> 75	2022-01-19 – 2022-06-30	115 (25)	21.74 (13.91 – 29.57)	0.631

Supplementary table 14: CFR for the glucocorticoid treatment group. CFR in percent for the glucocorticoid treatment group (cases where COVID-19 is the only indication excluded). The 95%CI for the CFR are estimated using a bootstrapping approach, the p-value is computed using a resampling procedure, and we employ the Benjamini-Hochberg procedure with an accepted FDR of 5% over all bins and treatment groups to account for multiple testing. ***Abbreviations:*** CFR: Case Fatality Rate.

2.15 Supplementary table 15: Glucocorticoid co-treatments

**Glucocorticoids, monotherapy cases**	**n reports**	**n died**	**n survived**
Prednisone	28	2	26
Prednisolone	7	1	6
Dexamethasone	3	0	3
Methylprednisolone	3	0	3
Dexamethasone;Prednisolone	1	0	1
Dexamethasone;Prednisone	1	0	1
	**n reports**	**n died**	**n survived**
**Glucocorticoids, co-treatments**			
Prednisone	811	131	680
Tacrolimus	525	81	444
Mycophenolate Mofetil	411	58	353
Dexamethasone	186	57	129
Hydroxychloroquine	173	15	158
Prednisolone	158	25	133
Mycophenolic Acid	118	26	92
Cyclophosphamide	99	39	60
Rituximab	95	29	66
Methotrexate	94	17	77
Methylprednisolone	76	12	64
Azithromycin Anhydrous	57	12	45
Azathioprine	54	8	46
Lenalidomide	54	18	36
Vincristine	44	9	35
Cyclosporine	41	5	36
Adalimumab	34	0	34
Tocilizumab	33	9	24
Daratumumab	32	11	21
Doxorubicin	32	9	23
Pomalidomide	31	10	21
Bortezomib	27	16	11
Mercaptopurine	27	1	26
Belatacept	26	10	16
Sirolimus	26	8	18
Carfilzomib	24	10	14
Thymocyte Immune Globulin Nos	23	1	22
Vincristine Sulfate	22	6	16
Cytarabine	19	4	15
Doxorubicin Hydrochloride	19	4	15
Lapine T-Lymphocyte Immune Globulin	19	2	17
Ruxolitinib	19	1	18
Remdesivir	17	7	10
Tofacitinib Citrate	16	4	12
Human Immunoglobulin G	15	2	13
Methylprednisolone Sodium Succinate	15	9	6
Albuterol Sulfate	14	4	10
Mycophenolate Sodium	14	2	12
Eculizumab	13	6	7
Basiliximab	12	1	11
Ceftriaxone	11	0	11
Abatacept	10	0	10
Budesonide	10	3	7
Docetaxel	10	0	10
Immune Globulin Nos	10	3	7
Acetaminophen	9	1	8

Apixaban	9	4	5
Covid-19 Vaccine Nos	9	1	8
Etoposide	9	1	8
Everolimus	9	0	9
Fluticasone Propionate	9	3	6
Sulfamethoxazole	9	3	6
Treprostinil	9	0	9
Trimethoprim	9	3	6
Betamethasone	8	0	8
Elotuzumab	8	4	4
Fludarabine Phosphate	8	5	3
Ipratropium Bromide	8	3	5
Ixazomib	8	0	8
Salmeterol Xinafoate	8	3	5
Venetoclax	8	1	7
Amoxicillin	7	2	5
Carboplatin	7	0	7
Dexamethasone Sodium Phosphate	7	5	2
Dupilumab	7	0	7
Methotrexate Sodium	7	2	5
Norepinephrine	7	1	6
Vancomycin	7	0	7
Abiraterone Acetate	6	0	6
Cefepime Hydrochloride	6	2	4
Ibrutinib	6	1	5
Oxaliplatin	6	5	1
Tofacitinib	6	2	4
Tozinameran	6	2	4
Amifampridine Phosphate	5	3	2
Asparaginase	5	0	5
Atorvastatin Calcium	5	1	4
Belimumab	5	0	5
Blinatumomab	5	1	4
Corticotropin	5	0	5
Leflunomide	5	0	5
Lopinavir	5	0	5
Meropenem	5	3	2
Piperacillin Sodium	5	0	5
Pyridostigmine	5	0	5
Ritonavir	5	0	5
Tazobactam Sodium	5	0	5
Anakinra	4	0	4
Bamlanivimab	4	3	1
Doxycycline Hyclate	4	0	4
Dulaglutide	4	0	4
Duloxetine Hydrochloride	4	0	4
Furosemide	4	0	4
Hydrochlorothiazide	4	0	4
Ibuprofen	4	0	4
Insulin Lispro	4	1	3
Ixazomib Citrate	4	1	3
Lenabasum	4	0	4
Lisinopril	4	0	4
Nivolumab	4	2	2
Norepinephrine Bitartrate	4	4	0
Obinutuzumab	4	0	4
Omeprazole	4	0	4

Oseltamivir	4	2	2
Pembrolizumab	4	2	2
Propofol	4	2	2
Selinexor	4	0	4
Sertraline Hydrochloride	4	0	4
Thioguanine	4	0	4
Ustekinumab	4	0	4
Abiraterone	3	0	3
Albuterol	3	2	1
Amlodipine Besylate	3	0	3
Beclomethasone Dipropionate	3	0	3
Bendamustine	3	1	2
Cholecalciferol	3	1	2
Ciprofloxacin Hydrochloride	3	0	3
Clopidogrel Bisulfate	3	0	3
Docusate Sodium	3	1	2
Elasomeran	3	2	1
Filgrastim	3	1	2
Gabapentin	3	1	2
Guaifenesin	3	0	3
Hydrocortisone	3	0	3
Hydroxychloroquine Sulfate	3	0	3
Insulin Glargine	3	0	3
Ipilimumab	3	0	3
Ipratropium	3	0	3
Leucovorin	3	0	3
Levetiracetam	3	0	3
Lorazepam	3	1	2
Losartan	3	0	3
Montelukast Sodium	3	0	3
Naloxone Hydrochloride	3	1	2
Nifedipine	3	0	3
Nitric Oxide	3	2	1
Ocrelizumab	3	0	3
Pantoprazole	3	0	3
Pantoprazole Sodium	3	1	2
Pegaspargase	3	2	1
Pyrimethamine	3	0	3
Sodium Chloride	3	1	2
Sulfadiazine	3	0	3
Upadacitinib	3	0	3
Acetylcysteine	2	2	0
Acyclovir	2	0	2
Ambrisentan	2	0	2
Apalutamide	2	0	2
Apremilast	2	0	2
Ascorbic Acid	2	1	1
Aspirin	2	1	1
Atovaquone	2	0	2
Azithromycin	2	0	2
Bleomycin Sulfate	2	0	2
Buprenorphine Hydrochloride	2	0	2
Cabotegravir	2	0	2
Casirivimab	2	0	2
Celecoxib	2	0	2
Chlorhexidine	2	0	2
Ciclesonide	2	0	2
Cisplatin	2	1	1

Codeine Phosphate	2	0	2
Covid-19 Convalescent Plasma	2	1	1
Dacarbazine	2	0	2
Daunorubicin	2	1	1
Dextromethorphan	2	0	2
Dextromethorphan Hydrobromide	2	0	2
Diclofenac Sodium	2	0	2
Dietary Supplement	2	0	2
Diphenhydramine Hydrochloride	2	0	2
Empagliflozin	2	0	2
Enoxaparin	2	0	2
Enoxaparin Sodium	2	1	1
Epcoritamab	2	2	0
Escitalopram Oxalate	2	0	2
Etesevimab	2	2	0
Famotidine	2	1	1
Fentanyl	2	0	2
Fentanyl Citrate	2	2	0
Ferrous Sulfate	2	0	2
Glucosamine	2	0	2
Heparin Sodium	2	0	2
Hydrocodone	2	0	2
Imdevimab	2	0	2
Infliximab	2	0	2
Lactulose	2	0	2
Leuprolide Acetate	2	0	2
Levofloxacin	2	0	2
Losartan Potassium	2	0	2
Melphalan	2	1	1
Mepolizumab	2	0	2
Midazolam	2	0	2
Nafamostat Mesylate	2	0	2
Nicotine	2	0	2
Nitazoxanide	2	0	2
Olanzapine	2	0	2
Paclitaxel	2	0	2
Panobinostat	2	1	1
Polyethylene Glycols	2	0	2
Procarbazine	2	0	2
Ribavirin	2	2	0
Rilpivirine	2	0	2
Riociguat	2	0	2
Rivaroxaban	2	2	0
Sodium Oxybate	2	0	2
Sodium Polystyrene Sulfonate	2	0	2
Tapentadol	2	2	0
Triamcinolone Acetonide	2	0	2
Ubidecarenone	2	0	2
Valganciclovir	2	0	2
Vinblastine	2	0	2
Vitamins	2	0	2
Zanamivir	2	2	0
Alemtuzumab	1	0	1
Alprazolam	1	0	1
Amikacin	1	0	1
Amiodarone	1	0	1
Amiodarone Hydrochloride	1	1	0

Amphotericin B	1	0	1
Belantamab Mafodotin	1	0	1
Belantamab Mafodotin-Blmf	1	0	1
Benzonatate	1	1	0
Betamethasone Dipropionate	1	0	1
Bumetanide	1	0	1
Buparlisib Hydrochloride	1	1	0
Busulfan	1	0	1
Calcium Carbonate	1	0	1
Ceftriaxone Sodium	1	0	1
Certolizumab Pegol	1	0	1
Chlorhexidine Gluconate	1	1	0
Ciltacabtagene Autoleucel	1	1	0
Cisatracurium	1	0	1
Citalopram Hydrobromide	1	1	0
Citarinostat	1	0	1
Clindamycin	1	0	1
Cortisone	1	0	1
Cyanocobalamin	1	0	1
Dapagliflozin Propanediol	1	0	1
Davoceticept	1	1	0
Denosumab	1	0	1
Dexmedetomidine Hydrochloride	1	1	0
Diltiazem Hydrochloride	1	0	1
Emapalumab	1	1	0
Entinostat	1	1	0
Epinephrine	1	0	1
Eptinezumab-Jjmr	1	0	1
Erythropoietin	1	1	0
Fingolimod Hydrochloride	1	0	1
Fluconazole	1	0	1
Flucytosine	1	0	1
Fluticasone Furoate	1	0	1
Folic Acid	1	0	1
Formoterol	1	0	1
Fosaprepitant Dimeglumine	1	0	1
Gemcitabine	1	1	0
Glucosamine Sulfate	1	0	1
Glycopyrrolate	1	0	1
Granulocyte Colony-Stimulating Factor Nos	1	0	1
Hyaluronidase-Fihj	1	0	1
Hydrocodone Bitartrate	1	0	1
Hydrocortisone Butyrate	1	0	1
Hydroxychloroquine Diphosphate	1	0	1
Hydroxyurea	1	0	1
Imatinib	1	0	1
Infliximab-Dyyb	1	0	1
Isatuximab	1	0	1
Ivermectin	1	0	1
Labetalol	1	0	1
Labetalol Hydrochloride	1	0	1
Lestaurtinib	1	0	1
Levothyroxine	1	0	1
Levothyroxine Sodium	1	1	0
Linezolid	1	0	1
Loratadine	1	1	0
Macitentan	1	0	1
Magnesium Chloride	1	1	0

Magnesium Hydroxide	1	0	1
Melatonin	1	1	0
Mesalamine	1	1	0
Metoprolol	1	0	1
Metronidazole	1	0	1
Mexiletine	1	0	1
Mexiletine Hydrochloride	1	1	0
Mosunetuzumab	1	1	0
Naloxone	1	0	1
Nintedanib	1	0	1
Niraparib	1	0	1
Nystatin	1	0	1
Omalizumab	1	0	1
Onabotulinumtoxina	1	0	1
Ondansetron Hydrochloride	1	1	0
Palbociclib	1	0	1
Palonosetron Hydrochloride	1	0	1
Penicillin	1	1	0
Polatuzumab Vedotin	1	1	0
Polyvinyl Alcohol	1	1	0
Potassium Chloride	1	1	0
Povidone	1	1	0
Primaquine Phosphate	1	0	1
Pyridostigmine Bromide	1	0	1
Pyridoxine	1	0	1
Radiation Therapy	1	0	1
Secukinumab	1	0	1
Semaglutide	1	0	1
Senna Leaf	1	1	0
Sennosides	1	1	0
Sennosides A And B	1	1	0
Sildenafil Citrate	1	0	1
Sodium Acetate	1	1	0
Sodium Gluconate	1	1	0
Spironolactone	1	0	1
Tadalafil	1	0	1
Tisagenlecleucel	1	1	0
Tobramycin	1	0	1
Umeclidinium Bromide	1	0	1
Unspecified Ingredient	1	1	0
Ursodiol	1	0	1
Valganciclovir Hydrochloride	1	0	1
Vancomycin Hydrochloride	1	0	1
Vasopressin	1	1	0
Vecuronium Bromide	1	1	0
Vilanterol Trifenatate	1	0	1
Vindesine	1	0	1
Voclosporin	1	0	1
Voxelotor	1	0	1
Zinc Sulfate	1	1	0

Supplementary table 15: Combination therapies in the glucocorticoid group. This table lists all cases where glucocorticoids are the only reported treatment with the respective report counts, and all treatments that are not in our glucocorticoid list and mentioned under *Suspect Product Active Ingredient* in a report considered for the glucocorticoid group with their respective report counts.

2.16 Supplementary table 16: CFR by vaccination coverage for the glucocorticoid group, without anti-CD20

Bin, % 1 dose	Covered period	n cases (n deaths)	CFR (95% CI)	p vs. baseline
baseline	2020-01-22 – 2020-12-12	355 (69)	19.44 (15.21 – 23.38)	–
0 – 20	2020-12-13 – 2021-03-06	159 (20)	12.58 (7.55 – 17.61)	0.071
5 – 25	2021-01-18 – 2021-03-16	94 (12)	12.77 (6.38 – 19.15)	0.176
10 – 30	2021-02-04 – 2021-03-25	101 (18)	17.82 (9.90 – 24.75)	0.862
15 – 35	2021-02-23 – 2021-04-04	68 (14)	20.59 (11.76 – 29.41)	0.944
20 – 40	2021-03-07 – 2021-04-13	60 (11)	18.33 (8.33 – 28.33)	0.998
25 – 45	2021-03-17 – 2021-04-27	50 (11)	22.00 (10.00 – 32.00)	0.782
30 – 50	2021-03-26 – 2021-05-18	44 (12)	27.27 (13.64 – 38.64)	0.304
35 – 55	2021-04-05 – 2021-06-28	77 (14)	18.18 (9.09 – 27.27)	0.934
40 – 60	2021-04-14 – 2021-08-13	155 (24)	15.48 (9.68 – 21.29)	0.360
45 – 65	2021-04-28 – 2021-10-03	201 (27)	13.43 (8.46 – 17.91)	0.093
50 – 70	2021-05-19 – 2021-11-25	311 (40)	12.86 (9.32 – 16.72)	0.033
55 – 75	2021-06-29 – 2022-01-18	330 (46)	13.94 (10.30 – 17.58)	0.071
> 60	2021-08-14 – 2022-06-30	349 (55)	15.76 (11.75 – 19.48)	0.237
> 65	2021-10-04 – 2022-06-30	295 (50)	16.95 (12.54 – 21.36)	0.476
> 70	2021-11-26 – 2022-06-30	164 (29)	17.68 (11.59 – 23.17)	0.719
> 75	2022-01-19 – 2022-06-30	106 (20)	18.87 (11.32 – 26.42)	> 0.9995

Supplementary table 16: CFR for the glucocorticoid treatment group, without anti-CD20 co-treatment. CFR in percent for all cases with glucocorticoid treatments, but without cases with anti-CD20 treatments as co-treatment (cases where COVID-19 is the only indication excluded). Note that the bin from 30%-50% does not fulfill our data selection criteria. The indicated bins are defined as (lower bound, upper bound], the 95% confidence intervals for the CFR are estimated using a bootstrapping approach, the p-value is computed using a resampling procedure, and we employ the Benjamini-Hochberg procedure with an accepted FDR of 5% over all bins and treatment groups to account for multiple testing. ***Abbreviations:*** CFR: Case Fatality Rate.

2.17 Supplementary table 17: CFR by vaccination coverage for JAK inhibitors

Bin, % 1 dose	Covered period	n cases (n deaths)	CFR (95% CI)	p vs. baseline
baseline	2020-01-22 – 2020-12-12	858 (42)	4.90 (3.50 – 6.41)	–
0 – 20	2020-12-13 – 2021-03-06	557 (43)	7.72 (5.57 – 9.87)	0.040
5 – 25	2021-01-18 – 2021-03-16	365 (32)	8.77 (6.03 – 11.78)	**0.013**
10 – 30	2021-02-04 – 2021-03-25	257 (15)	5.84 (3.11 – 8.67)	0.637
15 – 35	2021-02-23 – 2021-04-04	176 (11)	6.25 (2.84 – 10.23)	0.560
20 – 40	2021-03-07 – 2021-04-13	182 (10)	5.49 (2.20 – 8.79)	0.842
25 – 45	2021-03-17 – 2021-04-27	174 (12)	6.90 (3.45 – 10.92)	0.360
30 – 50	2021-03-26 – 2021-05-18	199 (16)	8.04 (4.52 – 12.06)	0.118
35 – 55	2021-04-05 – 2021-06-28	220 (15)	6.82 (3.64 – 10.45)	0.311
40 – 60	2021-04-14 – 2021-08-13	265 (18)	6.79 (3.77 – 9.81)	0.289
45 – 65	2021-04-28 – 2021-10-03	425 (24)	5.65 (3.53 – 7.76)	0.671
50 – 70	2021-05-19 – 2021-11-25	569 (34)	5.98 (4.04 – 7.91)	0.433
55 – 75	2021-06-29 – 2022-01-18	684 (46)	6.73 (4.82 – 8.63)	0.150
> 60	2021-08-14 – 2022-06-30	1710 (70)	4.09 (3.22 – 5.03)	0.407
> 65	2021-10-04 – 2022-06-30	1503 (59)	3.93 (2.99 – 4.92)	0.316
> 70	2021-11-26 – 2022-06-30	1302 (43)	3.30 (2.38 – 4.30)	0.082
> 75	2022-01-19 – 2022-06-30	1119 (30)	2.68 (1.79 – 3.66)	**0.013**

Supplementary table 17: CFR for the JAK inhibitors treatment group. CFR in percent for JAK inhibitors (only treatments from the JAK inhibitors group mentioned under *Suspect Product Active Ingredient*; cases where COVID-19 is the only indication excluded). The 95%CI for the CFR are estimated using a bootstrapping approach, the p-value is computed using a resampling procedure, and we employ the Benjamini-Hochberg procedure with an accepted FDR of 5% over all bins and treatment groups to account for multiple testing. ***Abbreviations:*** CFR: Case Fatality Rate.

2.18 Supplementary table 18: CFR by vaccination coverage for thalidomide analogs

Bin, % 1 dose	Covered period	n cases (n deaths)	CFR (95% CI)	p vs. baseline
baseline	2020-01-22 – 2020-12-12	802 (137)	17.08 (14.46 – 19.58)	-
0 – 20	2020-12-13 – 2021-03-06	809 (94)	11.62 (9.39 – 13.84)	**0.003**
5 – 25	2021-01-18 – 2021-03-16	486 (54)	11.11 (8.44 – 13.99)	**0.005**
10 – 30	2021-02-04 – 2021-03-25	330 (37)	11.21 (7.88 – 14.55)	**0.014**
15 – 35	2021-02-23 – 2021-04-04	207 (26)	12.56 (7.73 – 16.91)	0.137
20 – 40	2021-03-07 – 2021-04-13	160 (18)	11.25 (6.25 – 16.25)	0.092
25 – 45	2021-03-17 – 2021-04-27	158 (22)	13.92 (8.23 – 19.62)	0.407
30 – 50	2021-03-26 – 2021-05-18	160 (21)	13.12 (8.12 – 18.12)	0.284
35 – 55	2021-04-05 – 2021-06-28	206 (26)	12.62 (7.77 – 16.99)	0.141
40 – 60	2021-04-14 – 2021-08-13	261 (35)	13.41 (9.20 – 17.62)	0.191
45 – 65	2021-04-28 – 2021-10-03	497 (59)	11.87 (9.05 – 14.69)	**0.012**
50 – 70	2021-05-19 – 2021-11-25	714 (85)	11.90 (9.52 – 14.29)	**0.007**
55 – 75	2021-06-29 – 2022-01-18	1082 (97)	8.96 (7.30 – 10.63)	**< 0.001**
> 60	2021-08-14 – 2022-06-30	2275 (140)	6.15 (5.19 – 7.16)	**< 0.001**
> 65	2021-10-04 – 2022-06-30	1999 (107)	5.35 (4.40 – 6.35)	**< 0.001**
> 70	2021-11-26 – 2022-06-30	1734 (75)	4.33 (3.40 – 5.31)	**< 0.001**
> 75	2022-01-19 – 2022-06-30	1286 (55)	4.28 (3.19 – 5.37)	**< 0.001**

Supplementary table 18: CFR for the Thalidomide analogs treatment group. CFR in percent for thalidomide analogs (only treatments from the thalidomide analogs group mentioned under *Suspect Product Active Ingredient*; cases where COVID-19 is the only indication excluded). The 95% CI for the CFR are estimated using a bootstrapping approach, the p-value is computed using a resampling procedure, and we employ the Benjamini-Hochberg procedure with an accepted FDR of 5% over all bins and treatment groups to account for multiple testing. ***Abbreviations:*** CFR: Case Fatality Rate.

2.19 Supplementary table 19: COVID-19 reaction terms

FAERS reaction search term	n overall hits	n hits US only
Asymptomatic Covid-19	219	133
Breakthrough Covid-19	7	3
Congenital Covid-19	0	0
Coronavirus Infection	3325	2047
Coronavirus Pneumonia	64	26
Coronavirus Test Positive	512	339
Covid-19	51756	39931
Covid-19 Pneumonia	4362	2894
Covid-19 Treatment	1	1
Exposure To Sars-Cov-2	841	604
Occupational Exposure To Sars-Cov-2	6	6
Post-Acute Covid-19 Syndrome	129	86
Sars-Cov-2 Antibody Test Positive	169	126
Sars-Cov-2 Rna	4	0
Sars-Cov-2 Rna Increased	2	0
Sars-Cov-2 Sepsis	10	9
Sars-Cov-2 Test False Negative	8	5
Sars-Cov-2 Test Positive	5059	3940
Sars-Cov-2 Viraemia	7	1
Suspected Covid-19	2032	1046

Supplementary table 19: **COVID-19 related reaction search terms for querying FAERS.** All COVID-19 related *Reaction* search terms with hits in FAERS and the respective hit counts (overall, i.e. including hits before the first reported case in the respective country). ***Abbreviations:*** FAERS: FDA Adverse Event Reporting System.

2.20 Supplementary table 20: Treatments listed in ≥100 reports before and after campaign start

**Country**	***Suspect Product Active Ingredient***	**n reports**	**n baseline**	**n campaign**
US	Lenalidomide	3457	662	2795
US	Dupilumab	2836	335	2501
US	Tofacitinib Citrate	2541	675	1866
US	Adalimumab	2052	449	1603
US	Secukinumab	1398	394	1004
US	Human Immunoglobulin G	1295	569	726
US	Apixaban	1241	248	993
US	Ocrelizumab	1217	231	986
US	Pomalidomide	982	166	816
US	Valsartan	968	160	808
US	Sacubitril	967	160	807
CA	Infliximab	888	111	777
US	Prednisone	811	295	516
CA	Infliximab-Dyyb	806	192	614
US	Tacrolimus	801	330	471
GB	Adalimumab	774	448	326
US	Upadacitinib	758	123	635
US	Etanercept	644	157	487
US	Mycophenolate Mofetil	640	227	413
US	Palbociclib	564	148	416
US	Treprostinil	556	121	435
US	Apremilast	544	165	379
GB	Clozapine	408	218	190
US	Dimethyl Fumarate	349	118	231
US	Fingolimod Hydrochloride	347	108	239
US	Ustekinumab	324	109	215
US	Clozapine	273	107	166
BR	Secukinumab	252	128	124
US	Macitentan	234	112	122

Supplementary table 20: Treatments that met the minimal sample size required in the first filtering step. This table lists all treatments that were mentioned in at least 100 reports before the first vaccination dose was administered in the respective country (but after the first confirmed case) and in at least 100 reports after that date. Cases where COVID-19 is the only indication specified under *Reason for Use* were excluded. We built our treatment groups based on those treatments.

2.21 Supplementary table 21: Treatment groups

Treatment group	Included *Suspect Product Active Ingredient*
**Anti-CD20**	
	Ocrelizumab
	Ofatumumab
	Rituximab
	Rituximab-Abbs
	Rituximab-Arrx
	Rituximab-Pvvr
**Anti-TNFα**	
	Adalimumab
	Adalimumab-Adaz
	Adalimumab-Adbm
	Adalimumab-Afzb
	Adalimumab-Atto
	Adalimumab-Bwwd
	Adalimumab-Fkjp
	Certolizumab
	Certolizumab Pegol
	Etanercept
	Etanercept-Szzs
	Golimumab
	Infliximab
	Infliximab-Abda
	Infliximab-Axxq
	Infliximab-Dyyb
	Infliximab-Qbtx
**Glucocorticoids**	
	Betamethasone
	Betamethasone Sodium Phosphate
	Cortisone
	Cortisone Acetate
	Dexamethasone
	Dexamethasone Acetate
	Dexamethasone Sodium Phosphate
	Methylprednisolone
	Methylprednisolone Acetate
	Methylprednisolone Hemisuccinate
	Methylprednisolone Sodium Succinate
	Prednisolone
	Prednisolone Sodium Metazoate
	Prednisone
	Prednisone Acetate
**Dimethyl Fumarate**	
	Dimethyl Fumarate
**IL-17A blocker**	
	Brodalumab
	Ixekizumab
	Secukinumab
**IL-12, IL-23 blocker**	
	Guselkumab

	Risankizumab
	Risankizumab-Rzaa
	Ustekinumab
**IL-4, IL-13 blocker**	
	Dupilumab
**JAK inhibitors**	
	Baricitinib
	Ruxolitinib
	Ruxolitinib Phosphate
	Tofacitinib
	Tofacitinib Citrate
	Upadacitinib
**Thalidomide analogs**	
	Lenalidomide
	Pomalidomide
	Thalidomide
**Human Immunoglobulin G**	
	Human Immunoglobulin G
**Calcineurin inhibitors**	
	Tacrolimus
	Cyclosporine
**Factor Xa inhibitors**	
	Apixaban
	Betrixaban
	Edoxaban
	Rivaroxaban
**PDE4 inhibitors**	
	Apremilast
	Roflumilast
**Clozapine**	
	Clozapine
**Endothelin receptor antagonists**	
	Ambrisentan
	Atrasentan
	Bosentan
	Macitentan
	Sitaxentan
	Tezosentan
	Zibotentan
**Mycophenolate Mofetil**	
	Mycophenolate Mofetil
**CDK inhibitors**	
	Abemaciclib
	Palbociclib
	Ribociclib

Sacubitril and Valsartan	
	Sacubitril
	Valsartan

Prostacyclin analogs	
	Epoprostenol
	Iloprost
	Treprostinil

Supplementary table 21: Initial treatment groups. This table lists all treatment groups and the individual treatments included within each group for the initial filtering analysis, including biosimilars where applicable.

2.22 Supplementary table 22: CFR by vaccination coverage for the factor Xa inhibitors group

Bin, % 1 dose	Covered period	n cases (n deaths)	CFR (95% CI)	p vs. baseline
baseline	2020-01-22 – 2020-12-12	259 (22)	8.49 (5.02 – 11.97)	-
0 – 20	2020-12-13 – 2021-03-06	320 (39)	12.19 (8.75 – 15.94)	0.198
5 – 25	2021-01-18 – 2021-03-16	222 (19)	8.56 (4.95 – 12.16)	> 0.9995
10 – 30	2021-02-04 – 2021-03-25	165 (15)	9.09 (4.85 – 13.33)	0.955
15 – 35	2021-02-23 – 2021-04-04	112 (8)	7.14 (2.68 – 11.61)	0.825
20 – 40	2021-03-07 – 2021-04-13	89 (5)	5.62 (1.12 – 10.11)	0.531
25 – 45	2021-03-17 – 2021-04-27	91 (6)	6.59 (2.20 – 12.09)	0.755
30 – 50	2021-03-26 – 2021-05-18	113 (6)	5.31 (1.77 – 9.73)	0.385
35 – 55	2021-04-05 – 2021-06-28	162 (10)	6.17 (2.47 – 9.88)	0.496
40 – 60	2021-04-14 – 2021-08-13	204 (17)	8.33 (4.90 – 12.25)	> 0.9995
45 – 65	2021-04-28 – 2021-10-03	260 (26)	10.00 (6.54 – 13.85)	0.655
50 – 70	2021-05-19 – 2021-11-25	276 (37)	13.41 (9.42 – 17.39)	0.094
55 – 75	2021-06-29 – 2022-01-18	274 (48)	17.52 (12.77 – 22.26)	**0.004**
> 60	2021-08-14 – 2022-06-30	395 (57)	14.43 (10.89 – 17.72)	0.030
> 65	2021-10-04 – 2022-06-30	310 (46)	14.84 (10.97 – 18.71)	0.027
> 70	2021-11-26 – 2022-06-30	249 (34)	13.65 (9.24 – 18.07)	0.085
> 75	2022-01-19 – 2022-06-30	185 (19)	10.27 (5.95 – 14.59)	0.628

Supplementary table 22: CFR for the factor Xa inhibitors treatment group, US. CFR in percent computed for the factor Xa inhibitors treatment group (only treatments from the factor Xa inhibitors group mentioned under *Suspect Product Active Ingredient*; cases where COVID-19 is the only indication excluded). The indicated bins are defined as (lower bound, upper bound], the 95% confidence intervals for the CFR are estimated using a bootstrapping approach, the p-value is computed using a resampling procedure, and we employ the Benjamini-Hochberg procedure with an accepted FDR of 5% over all bins and treatment groups to account for multiple testing.
Abbreviations: CFR: Case Fatality Rate.

3 Supplementary Figures

3.1 Supplementary figure 1: CFR by vaccination coverage in OWID

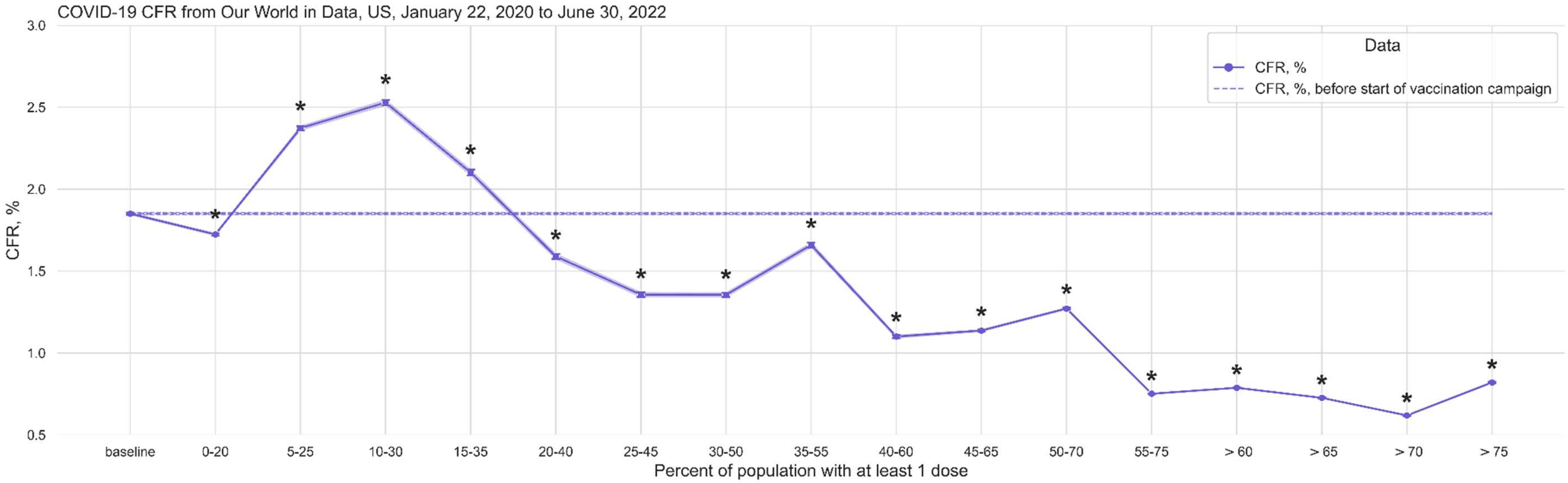

Supplementary figure 1: CFR by vaccination coverage in OWID. CFR due to COVID-19 from OWID, US. CFR from OWID data for the US during the baseline period (first bin, and dashed line with 95% confidence interval drawn over the entire plot range for reference) and for 20% bins in vaccination coverage (at least one dose). Bins are indicated as (lower bound, upper bound], and the baseline period is defined as the period before the first vaccination was administered. Since a coverage of 80% has not yet been achieved at the time of data collection, we denote bins starting from 60% or higher coverage as "> x", indicating that these bins include all data from coverage levels higher than x. The 95% confidence intervals are the Wilson's score intervals. Asterisks mark data points where the CFR in the bin is significantly different from the baseline value (G-test). ***Abbreviations*****:** CFR: Case Fatality Rate; COVID-19: Corona Virus Disease-2019; OWID: Our World In Data.

3.2 Supplementary figure 2: Cumulative COVID-19 cases and deaths from OWID and FAERS data

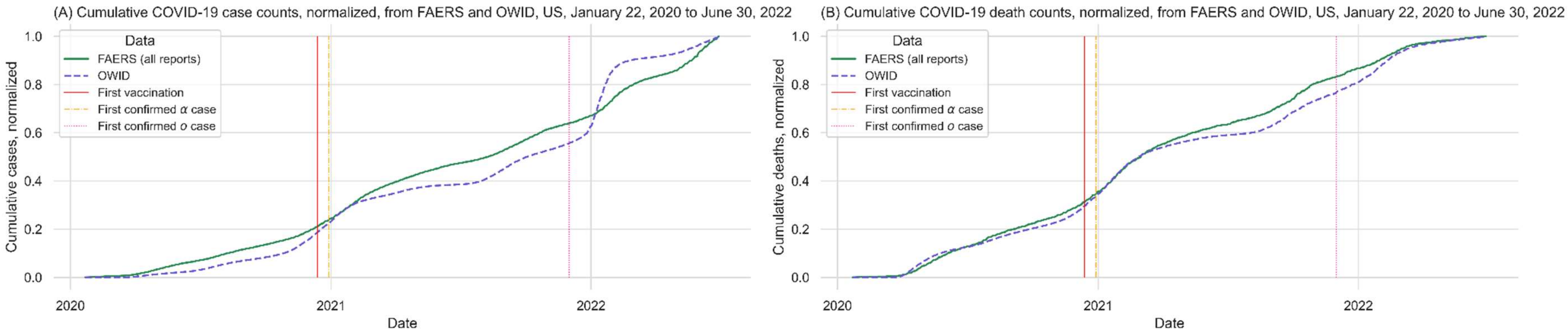

Supplementary figure 2: Cumulative COVID-19 cases and deaths in OWID and FAERS. Cumulative COVID-19 case and death counts in OWID and FAERS. ***(A)*** Cumulative case counts, normalized to [0, 1], from OWID data (blue dashed line) and the FAERS COVID-19 dataset (all treatments and indications, green solid line), for the US. ***(B)*** Cumulative death counts, normalized to [0, 1], from OWID data (blue dashed line) and the FAERS COVID-19 dataset (all treatments and indications, green solid line), for the US. The first vaccination was administered on December 13, 2020 (vertical solid red line), the first Alpha variant case was confirmed on December 29, 2020 (vertical dot-dashed orange line), and the first Omicron variant case was confirmed on December 1, 2021 (vertical dotted pink line). We observe that variant peaks, especially the omicron peak, are not as pronounced in FAERS as they are in OWID. This is partly due to the inherent limitation of using self-reported cases, where cases are often reported with a delay. ***Abbreviations:*** FAERS: FDA Adverse Event Reporting System; OWID: Our World In Data.

3.3 Supplementary figure 3: CFR by vaccination coverage for glucocorticoids with and without anti-CD20

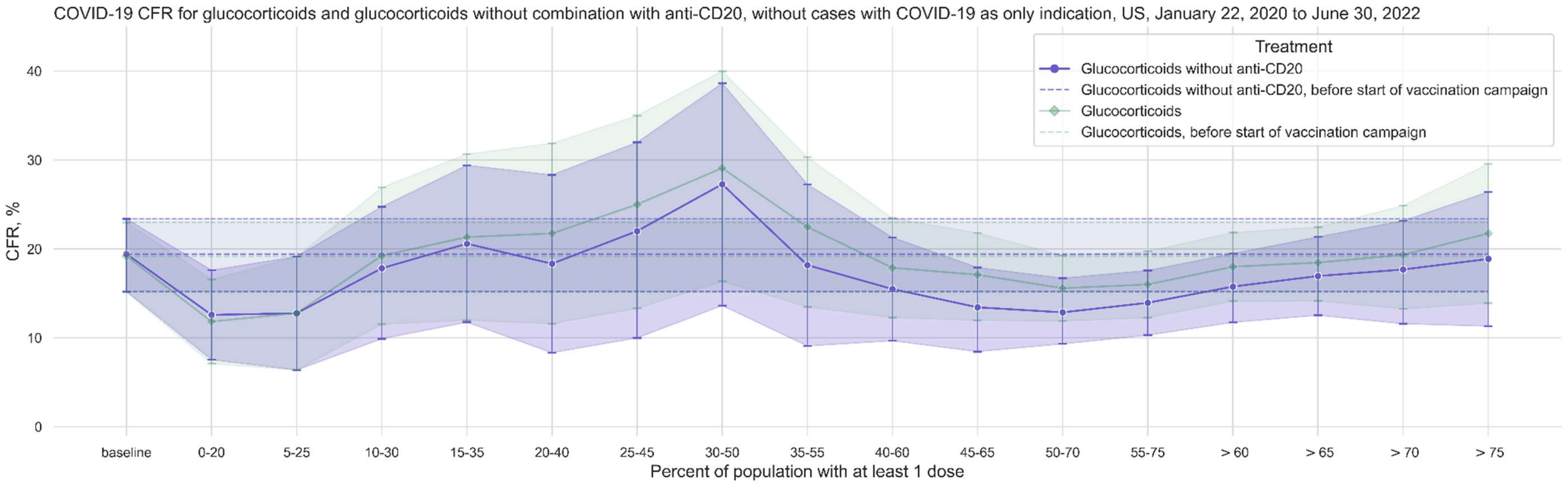

Supplementary figure 3: COVID-19 CFR by vaccination coverage for glucocorticoids with and without anti-CD20. CFR for the glucocorticoid group with cases with anti-CD20 co-treatment removed (cases where COVID-19 is the only indication excluded) (shown in blue) and the original glucocorticoid group (shown in green), US only, in 20% vaccination coverage bins. Bins are indicated as (lower bound, upper bound], and the baseline period is defined as the period before the first vaccination was administered. Since a coverage of 80% had not yet been achieved at the time of data collection, we denote bins starting from 60% or higher coverage as "> x", indicating that these bins include all data from coverage levels higher than x. The 95% confidence intervals are estimated using bootstrap resampling, and asterisks mark data points where the CFR in the glucocorticoids without anti-CD20 group is significantly different from the CFR during the baseline period for this group (p-value from resampling, Benjamini-Hochberg with an accepted FDR of 5% over all bins and treatment groups). ***Abbreviations:*** CFR: Case Fatality Rate; FAERS: FDA Adverse Event Reporting System; OWID: Our World In Data; sig.: Significant.

3.4 Supplementary figure 4: CFR by vaccination coverage for the factor Xa inhibitors group

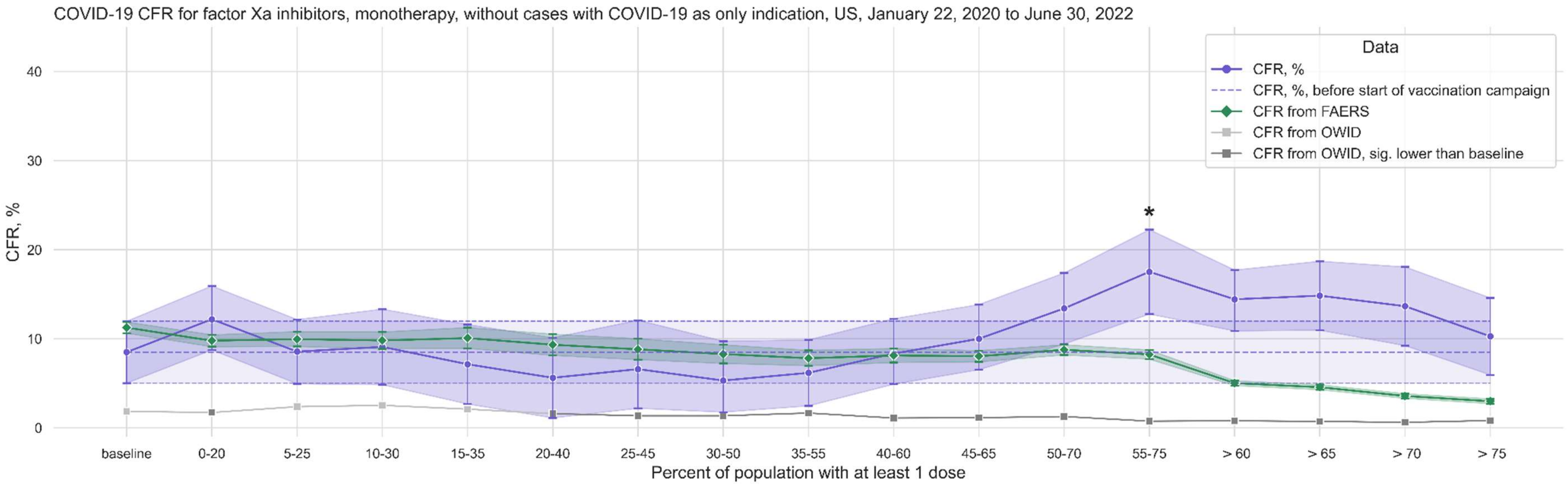

Supplementary figure 4: COVID-19 CFR by vaccination coverage for the factor Xa inhibitors group. CFR for the factor Xa inhibitors treatment group (only treatments from the factor Xa inhibitors group mentioned under *Suspect Product Active Ingredient*; cases where COVID-19 is the only indication excluded) (shown in blue), US only, in 20% vaccination coverage bins. Bins are indicated as (lower bound, upper bound], and the baseline period is defined as the period before the first vaccination was administered. Since a coverage of 80% had not yet been achieved at the time of data collection, we denote bins starting from 60% or higher coverage as "> x", indicating that these bins include all data from coverage levels higher than x. The 95% confidence intervals are estimated using bootstrap resampling, and asterisks mark data points where the CFR in the treatment group is significantly different from the CFR during the baseline period for this group (p-value from resampling, Benjamini-Hochberg with an accepted FDR of 5% over all bins and treatment groups. Data for the complete FAERS set are shown in green (note that significant data points are not annotated for better readability; see Figure 1A for FAERS details). The grey line shows the CFR for OWID data, with data points where the CFR is significantly lower than during the baseline period shown in a darker grey. ***Abbreviations:*** CFR: Case Fatality Rate; FAERS: FDA Adverse Event Reporting System; OWID: Our World In Data; sig.: Significant.

4 References

1. **U.S. Food & Drug Administration (FDA).** FDA Adverse Event Reporting System. Oct 22, 2012. https://www.fda.gov/drugs/questions-and-answers-fdas-adverse-event-reporting-system-faers/fda-adverse-event-reporting-system-faers-public-dashboard (data downloaded Aug 12, 2022).

2. **Mathieu E, Ritchie H, Rodés-Guirao L, et al.** Coronavirus Pandemic (COVID-19). *Our World in Data 2020*. https://ourworldindata.org/coronavirus (data downloaded Aug 12, 2022).

3. **U.S. National Institutes of Health COVID-19 Treatment Guidelines Panel.** Coronavirus Disease 2019 (COVID-19) Treatment Guidelines. Aug 8, 2022. https://www.covid19treatmentguidelines.nih.gov/ (accessed Mar 3, 2023).
